# Action and cooperation in alginate degradation by three enzymes from the human gut bacterium *Bacteroides eggerthii* DSM 20697

**DOI:** 10.1016/j.jbc.2024.107596

**Published:** 2024-07-19

**Authors:** Mette E. Rønne, Christian Dybdahl Andersen, David Teze, Agnes Beenfeldt Petersen, Folmer Fredslund, Emil G.P. Stender, Evan Kirk Chaberski, Jesper Holck, Finn L. Aachmann, Ditte Hededam Welner, Birte Svensson

**Affiliations:** 1 Enzyme and Protein Chemistry, Department of Biotechnology and Biomedicine, Technical University of Denmark, Lyngby, Denmark; 2 Norwegian Biopolymer Laboratory (NOBIPOL), Department of Biotechnology and Food Science, NTNU Norwegian University of Science and Technology, Trondheim, Norway; 3 Enzyme Engineering and Structural Biology, Novo Nordisk Foundation Center for Biosustainability, Technical University of Denmark, Lyngby, Denmark; 4 Enzyme Technology, Department of Biotechnology and Biomedicine, Technical University of Denmark, Lyngby, Denmark

**Keywords:** alginate, alginate lyase, KdgF-like enzyme, carbohydrate processing, enzyme kinetics, enzyme mechanism, human gut microbiota, polysaccharide utilization locus, protein structure

## Abstract

Alginate is a polysaccharide consumed by humans in edible seaweed and different foods where it is applied as a texturizing hydrocolloid or in encapsulations of drugs and probiotics. While gut bacteria are found to utilize and ferment alginate to health-beneficial short-chain fatty acids, knowledge on the details of the molecular reactions is sparse. Alginates are composed of mannuronic acid (M) and its C-5 epimer guluronic acid (G). An alginate-related polysaccharide utilization locus (PUL) has been identified in the gut bacterium *Bacteroides eggerthii* DSM 20697. The PUL encodes two polysaccharide lyases (PLs) from the PL6 (*Be*PL6) and PL17 (*Be*PL17) families as well as a KdgF-like metalloprotein (*Be*KdgF) known to catalyze ring-opening of 4,5-unsaturated monouronates yielding 4-deoxy-l-*erythro*-5-hexoseulose uronate (DEH). *B. eggerthii* DSM 20697 does not grow on alginate, but readily proliferates with a lag phase of a few hours in the presence of an *endo*-acting alginate lyase A1-I from the marine bacterium *Sphingomonas* sp. A1. The *B. eggerthii* lyases are both *exo*-acting and while *Be*PL6 is strictly G-block specific, *Be*PL17 prefers M-blocks. *Be*KdgF retained 10−27% activity in the presence of 0.1−1 mM EDTA. X-ray crystallography was used to investigate the three-dimensional structure of *Be*KdgF, based on which a catalytic mechanism was proposed to involve Asp102, acting as acid/base having p*K*_a_ of 5.9 as determined by NMR pH titration. *Be*PL6 and *Be*PL17 cooperate in alginate degradation with *Be*KdgF linearizing producing 4,5-unsaturated monouronates. Their efficiency of alginate degradation was much enhanced by the addition of the A1-I alginate lyase.

Further understanding of the molecular mechanisms associated with the consumption of marine foods, like seaweed, is widely sought (1, 2). This study focuses on alginate and the role of bacterial enzymes in facilitating its digestion in the human gut (3, 4). Alginates are anionic, linear polysaccharides composed of 1,4-linked β-d-mannuronate (M) and its C-5 epimer α-l-guluronate (G), that occur in cell walls of brown algae and constitute 30−60% of the dry weight (5, 6). The uronic acid residues are arranged in blocks of M, G, or mixed MG with M being predominantly in the ^4^C_1_ and G in the ^1^C_4_ conformation (Fig. 1) (7, 8). Alginates can form gels at low pH as well as in the presence of divalent cations like Ca^2+^ and serve as viscosifying, stabilizing, encapsulating, and hydrogel-forming agents in food, technical, and pharmaceutical industries (6, 9, 10, 11). Notably, some bacteria produce alginate as part of their biofilm, *e.g.* the pathogenic *Pseudomonas aeruginosa* (12).

**Figure 1:**
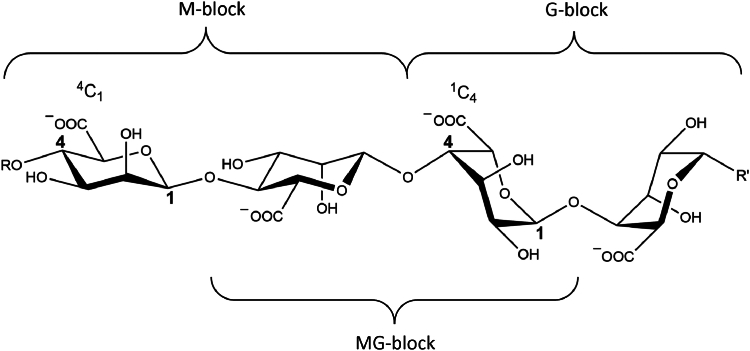
**Structure of alginate.** β-d-mannuronate (M) and its C-5 epimer α-l-guluronate (G) are making up alginate arranged in blocks of M, G and MG residues.

Enzymes encoded by the human genome are not able to degrade alginate. However, certain species of the *Bacteroides* genus, including *Bacteroides ovatus* and *Bacteroides xylanisolvens* of the human gut microbiota (HGM) are capable of depolymerizing alginates to oligosaccharides (AOSs) (13) and members of the HGM were found *in vitro* to further degrade and metabolize AOSs with the formation of health beneficial short-chain fatty acids (SCFA) (4, 14, 15, 16). Moreover, AOSs are reported to be bifidogenic and promote the proliferation of different *Bifidobacterium* species (14). This ability motivated the interrogation of alginates as an emerging prebiotic dietary fiber contributing to a healthy gut environment (14, 17, 18). Structure and function have been reported for a small number of alginate-degrading enzymes of HGM origin, including BACEGG_03249 from *Bacteroides eggerthii* DSM 20697, which is now more thoroughly described here and named *Be*PL17 (19, 20, 21, 22, 23, 24). By contrast, several alginolytic enzymes from marine bacteria, including members of the *Bacteroidetes* phylum have been studied in detail (19, 25, 26, 27, 28, 29). *Bacteroides* is a genus of Gram-negative, obligate anaerobic bacteria and among the most abundant genera in the HGM belonging to *Bacteroidetes* that together with the *Firmicutes* phylum of Gram-positive bacteria accounts for about 90% of the gut microbiota (30). *Bacteroides* can grow on a wide range of polysaccharides (31, 32, 33) and contain distinct gene clusters, referred to as Polysaccharide Utilization Loci (PULs), that encode enzymes, binding proteins, regulators, and transporters dedicated to the utilization of a large variety of specific carbohydrates (19, 34, 35). PULs are recognized by the presence of genes homologous to the SusC (SusC_H_) transporter and SusD (SusD_H_) binding protein first identified in the locus for the Starch Utilization System in *Bacteroides thetaiotaomicron* (36). Predicted and characterized PULs from *Bacteroidetes* are organized in the PUL database (PULDB) (37, 38) depicting sequences and putative functions of the encoded proteins, including several Carbohydrate Active enZymes (CAZymes) (39, 40). The marine flavobacterium *Zobellia galactanivorans* DsijT has a PUL structure similar to *Bacteroides* strains (41) containing an extracellular *endo*-acting alginate lyase (AlyA1) and it can grow on alginate as sole carbon source (42). Such PULs are also found in the marine Bacteroidetes *Gramella forsetii* KT0803 (43), and in other alginate-utilizing marine bacteria including *Agarivorans* sp. B2Z047, *Pseudoalteromonas* (29, 44), and *Sphingomonas* sp. strain A1 (45, 46). A short PUL encoding six genes including of two predicted alginate lyases has been reported before for the gut bacterium *B. eggerthii* DSM 20697 (19). Here, this PUL is considered a shorter version of the longer PUL described in the present study.

Alginate depolymerizing enzymes are all lyases and found in several of the 43 polysaccharide lyase (PL) families in the CAZy database (www.cazy.org), altogether degrading uronic acid-containing polysaccharides, such as pectin, polygalacturonate, polyglucuronate, chondroitin, xanthan, heparin, alginate and hyaluronate (47). Some PL families are multispecific and to date 14 of the 43 PL families are indicated in the CAZy database (39) to encompass alginate lyases, *i.e.* PL5, 6, 7, 8, 14, 15, 17, 18, 31, 32, 34, 36, 39 and 41. In addition, two alginate degrading lyases have been reported recently to belong to family PL38 (24, 44). These enzymes catalyze cleavage of alginates in a β-elimination reaction leading to products having a 4,5-unsaturated bond in the non-reducing end residue, denoted as Δ. Alginate lyases can be *endo*- or *exo*-acting, the latter releasing 4,5-unsaturated monouronate (4-deoxy-β-L-*threo*-hex-4-enopyranuronic acid), referred to as Δ, from either alginate or AOSs, which have been produced by *endo*-acting enzymes (19, 23, 43, 48). Some alginate lyases specifically act on either M-M (49, 50), G-G (25, 51), M-G or G-M linkages (52, 53), while others are able to cleave more than one of these specific linkages (8, 24, 54). Additionally, alginate lyases can prefer polysaccharides or oligosaccharide substrates (26). As opposed to the AOS products having Δ at the non-reducing end, monomeric Δ undergoes spontaneous ring-opening (linearization) and tautomerization to 4-deoxy-l-*erythro*-5-hexoseulose uronate (DEH) (8, 25, 28, 55, 56). This reaction has also previously been shown to be catalyzed by KdgF-like enzymes from *Yersinia enterocolitica* and *Halomonas* sp. acting on Δ generated from dimannuronate by an alginate lyase of PL17 (55). Next DEH undergoes hydration and further change to multiple products in equilibrium, predominantly the two 5-membered rings 4-deoxy-d-*manno*-hexulofuranosidonate (DHF) (5*S*)-DHF hydrate and (5*R*)-DHF hydrate (8).

While numerous genes encoding putative alginate lyases have been identified in bacteria of the HGM (www.cazy.org/PULDB) (38, 39), only seven bacterial alginate lyases were characterized from this niche (23), including PL6 members *Bcel*PL6 and *Bc*AlyPL6 from *Bacteroides cellulosilyticus* CRE21 (20) and *Bacteroides clarus* (22), as well as a PL17 (BACEGG_03249) from *B. eggerthii* DSM 20697 (19). The specificity and mode of action of the latter enzyme were characterized in a phylogenetic coverage mapping endeavor on family PL17 (19). Moreover, a PL32 enzyme from *Bacteroides salyersiae* was identified in a screening of uncharacterized CAZymes for function (21). Very recently, three alginate lyases, *Bo*PL6, *Bo*PL17 and *Bo*PL38, were identified and characterized by us from the HGM *B. ovatus* CP926, which intriguingly was able to grow on alginate as the sole carbon source (24). *Bcel*PL6, that produces mainly di- and trisaccharides from M-blocks in alginate (20) is not annotated to a PUL and *B. cellulosilyticus* does not encode other putative alginate lyases (20). Two *exo*-acting enzymes belonging to PULs are able to release Δ, BACEGG_03249, a PL17 member acting on polyM (19), and *Bc*AlyPL6 acting on both polyG and polyMG (22). Additional evidence for alginate utilization by HGM stems from the markedly increased relative abundance of *Faecalibacterium prausnitzii* in human fecal samples by growth on alginate, presumably due to cross-feeding as this bacterium did not grow on alginate in monoculture (4). Further, a few isolated *Bacteroidetes* strains from HGM were recently shown to degrade alginate, suggested to reflect gene transfer from marine to gut bacteria associated with the digestion of edible seaweed (13).

To contribute new insights on molecular reactions occurring in the gut by human consumption of seaweeds, here three alginate degrading enzymes encoded by a PUL containing 10 genes identified in *B. eggerthii* of the HGM were produced recombinantly and characterized. A version of this PUL was reported to comprise six genes, including for alginate lyases of PL6 and PL17, and similar short PULs were seen in the human gut bacteria *Bacteroides* sp. *1_1_30* and *Bacteroides* sp. *D2* (19). Although *B. eggerthii* did not grow on alginate, it could utilize AOSs formed by the *endo*-acting alginate lyase A1-I from *Sphingomonas* sp. A1 (46, 57) added to the culture medium. Here we produced and characterized *B. eggerthii Be*PL6 and *Be*PL17 and show that they cooperate and release Δ from alginate, which in turn was linearized by *Be*KdgF encoded by the same PUL. The crystal structure, p*K*_a_ of a proposed catalytic acid/base, enzyme kinetics, role of pH and metal ions on the activity and stability of *Be*KdgF expanded knowledge on this scarcely studied large enzyme family.

## Results

### Identification of alginate degrading enzymes in a human gut bacterial genome

**Figure 2:**
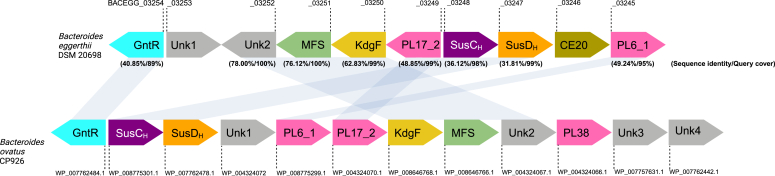
**Polysaccharide utilization loci from *Bacteroides eggerthii* DSM 20697 and *Bacteroides ovatus* CP926**. The *B. eggerthii* PUL (*Top*) appears to be involved in alginate metabolism. It spans 10 genes (Locus tags BACEGG_03245–03254) predicted to encode a characteristic SusC_H_/SusD_H_ complex for carbohydrate binding and transport, two alginate lyases of PL6 subfamily 1 (PL6_1) and PL17 subfamily 2 (PL17_2) and a KdgF-like enzyme (annotated as Unk3) in the PULDB. For the *B. eggerthii* PUL Locus tags are given above each gene according to the PULDB (see also Table 1). Underneath the proteins are indicated the sequence identity with homologous proteins from *B. ovatus* CP926 (*Bottom*) for which GenBank accession numbers listed under each gene. Homologous genes are connected by *gray* bars.

Gene-mining of the PULDB (37) for alginate utilization identified a predicted PUL in *B. eggerthii* DSM 20967 (onwards referred to as *B. eggerthii*) (Fig. 2) encoding proteins involved in polysaccharide utilization, *i.e.* polysaccharide lyases of families PL6 and PL17 − supposedly acting on alginate, putative homologs of SusC (SusC_H_) and SusD (SusD_H_) (35, 37, 38), a major facilitator superfamily (MFS) transporter, a GntR transcription regulator, a carbohydrate esterase of family 20 (CE20) and four genes marked as of unknown function, Unk1−Unk4. The Unk3 gene encodes a homolog of the KdgF-like enzymes from *Y. enterocolitica* and *Halomonas* sp. catalyzing linearization of Δ released by polysaccharide lyases from pectin and alginate (55). The closest BLAST hits against the UniProt database of the genes in the *B. eggerthii* PUL are listed in Table 1. As mentioned above a shorter version of this PUL was previously described to encode six (KdgF, PL17_2, SusC_H_, SusD_H_, Unk4/CE20 and PL6_1) of the 10 proteins displayed in Figure 2 and the PL17 member was reported to be polyM specific and *exo*-acting (19). The two predicted alginate lyases belong to the most common subfamilies 1 and 2 of PL6 and PL17, respectively (19, 27). As also informed by the BLAST search family PL6 contains a small number of chondroitin lyases, first identified in this family, but according to the CAZy database (39) the vast majority of characterized PL6 members are alginate lyases. *Be*PL6 and *Be*PL17, as well as SusC_H_, SusD_H,_ and CE20, contain signal peptides according to SignalP (58) and are assumed to be secreted to the periplasmic space or the outer membrane. The three predicted proteins MFS, SusC_H,_ and Unk4 contain transmembrane segments. While Unk3 was identified as *Be*KdgF, homologs of the Unk1, Unk2, and Unk4 proteins were not assigned a putative function in the PULDB, but showed > 40% sequence similarities with two reductases and a carbohydrate esterase of family CE20 (38, 39) (Table 1). A CE20 esterase from a plant pathogenic bacterium was recently reported to catalyze deacetylation of xyloglucan (59), which is considered a dietary fiber.

**Table 1:** Genes encoded by a PUL in *Bacteroides eggerthii* DSM 20697

Modularity	Signal peptide	Transmembrane domain	UniProt accession	Putative function of closest hit	Identity (%)	E-value
GntR	No	No	A0A7W5DQC8	Transcriptional repressor for pyruvate dehydrogenase	53.7	2.7e-78
Unk 1	No	No	E4T4B2	Glyoxylate reductase	52.2	1.9e-109
Unk 2	No	No	A0A2V3PVV	Oxidoreductase	77.2	1e-136
MFS	No	Yes	A0A2V3PT66	Transmembrane transporter	75.2	0.0
Unk 3/KdgF	No	No	A0A374VDY6	Cupin domain protein	71.2	2.7e-55
PL17_2	Yes	No	A0A2V3PV97	Exolytic alginate lyase	55.3	0.0
SusC_H_	Yes	Yes	G8R4Q8	Ton-dependent receptor SusC_H_	44.6	0.0
SusD_H_	Yes	No	G8R4Q9	SusD_H_ binding protein	38.6	2.7e-87
Unk 4/CE20	Yes	Yes	A0A286RHI4	Sialate *O*-acetylesterase	41.2	9.8e-127
PL6_1	Yes	No	A0A174F121	Chondroitin B lyase	48.6	3e-80

Putative functions are indicated of the 10 proteins encoded by the alginate degradation PUL in *B. eggerthii* using BLAST (110).

Several other PULs also contain genes encoding PL6, PL17, and KdgF; however, KdgF enzymes are not annotated explicitly in the PULDB at present (37). The recently identified new isolate *B. ovatus* CP926 (24), which is currently not in PULDB, contains such a PUL (Fig. 2). A known hallmark of PULs is the transporter complex of SusC_H_ and SusD_H_, present in the *B. eggerthii* and *B. ovatus* CP926 PULs, in addition to GntR, an FadR family transcriptional regulator. SusC_H_ and SusD_H_ are co-localized in the PULs and show 36.12% and 31.81% sequence identity, respectively (Fig. 2), while the GntR regulators have 40.85% sequence identity. KdgF, MFS, and Unk2 are co-localized in both bacteria in this order with sequence identities of 62.83%, 76.12%, and 78.00%, respectively. The two bacteria encode polysaccharide lyases, shown to act on alginate, of subfamilies PL6_1 with 49.24% and PL17_2 with 48.85% sequence identity (39). However, these PL genes are co-localized only in *B. ovatus* CP926. Notably, the PUL of *B. ovatus* CP926 encodes two more proteins than the *B. eggerthii* PUL, namely a multi-specific PL38 alginate lyase (24) and a protein of unknown function with no identified homologs.

### *B. eggerthii* grows on AOSs produced *in situ* by a marine *endo*-acting alginate lyase

**Figure 3:**
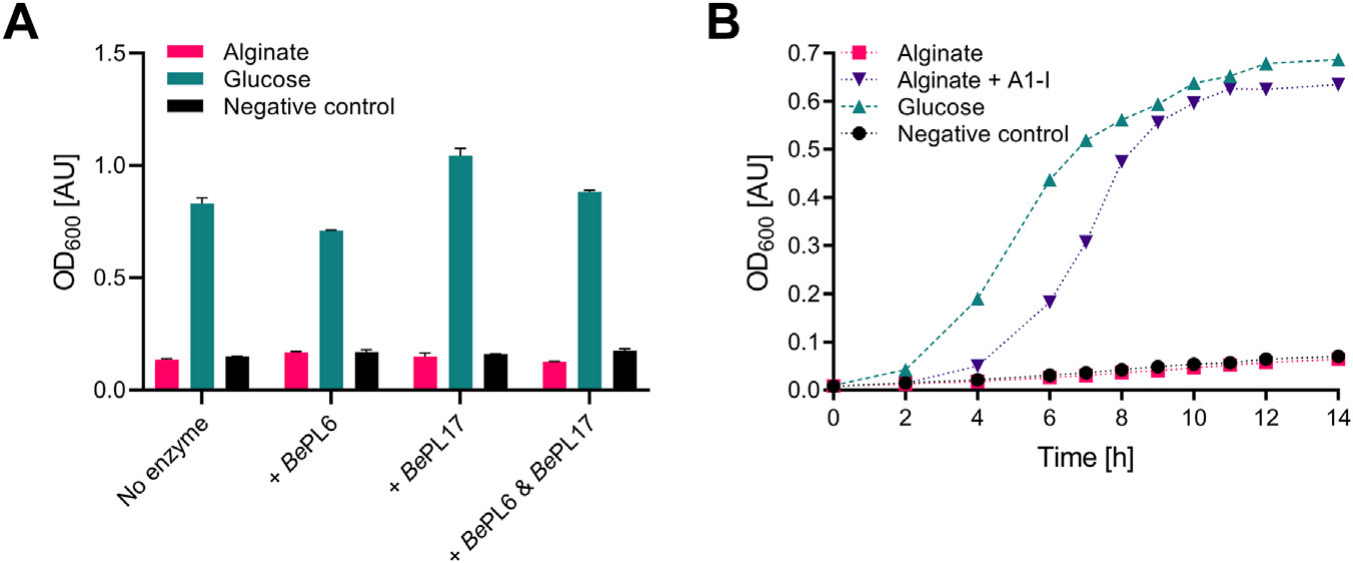
**Growth of *Bacteroides eggerthii* DSM 20697**. *A*, culture (37 °C, O/N) on 0.5% alginate or 0.5% glucose without and with 100 nM *Be*PL6 and *Be*PL17 either added separately or in combination. *B*, growth on 0.5% alginate without and with added 100 nM A1-I from *Sphingomonas* sp. A1-I. Cultures on 0.5% glucose and without carbon source serve as positive and negative controls, respectively. Growth (in triplicate) measured at 600 nm was plotted as the mean with standard deviation (for several measurements error bars and mean overlap).

*B. eggerthii* neither grew on alginate as sole carbon source nor when its own two recombinantly produced alginate lyases *Be*PL6 and *Be*PL17 were added to the culture medium (Fig. 3*A*). Certain bacteria encode a presumed secreted *endo*-acting alginate lyase releasing AOSs transported *via* the SusC_H_/SusD_H_ complex into the periplasmic space (60). However, *B. eggerthii* DSM 20697 seems to lack an extracellular *endo*-acting alginate lyase and probably relies on AOSs produced by primary alginate degraders in the HGM (24). Indeed, the addition of the very efficient *endo*-acting A1-I from the marine bacterium *Sphingomonas* sp. strain A1 (46, 57), which produces mainly AOSs of DP2-4 (Fig. S1), enabled excellent growth of *B. eggerthii* on alginate as sole carbon source (Fig. 3*B*). Notably, this culture, even though the lag-phase was longer than on glucose, resulted in comparable OD_600_ values after 9 h (Fig. 3*B*) and was fully utilizing the substrate.

### Recombinant production and biochemical characterization of *Be*PL6 and *Be*PL17 alginate lyases

About 50% of the family PL6 members possess two parallel right-handed β-helix domains of which the N-terminal corresponds to the remaining about 50% of one-domain PL6 members and carries the active site. *Be*PL6 is a two-domain enzyme (39). The role of the C-terminal domain is not known (25), but it has been proposed to be important for substrate accessibility and binding to the active site (22). The PL17 enzymes have an (α/α)_6_ toroid + anti-parallel β-sheet fold, of which the (α/α)_6_ toroid domain contains the active site (11, 61). Recombinant production resulted in good yields for both *Be*PL6 (21 mg L^−1^) and *Be*PL17 (19 mg L^−1^). *Be*PL6 and *Be*PL17 migrated as single bands of about 80 and 75 kDa in SDS-PAGE (Fig. S2), corresponding to theoretical molecular masses as predicted by ProtParam (62) of 84,151 and 82,094 Da, respectively. Elution by size exclusion chromatography on HiLoad Superdex 200 pg 16/600 indicated that *Be*PL6 is a monomer and *Be*PL17 a dimer in solution (data not shown). Other PL17 enzymes are also shown to be dimers in solution (28, 63). However, PL6 enzymes differ, thus *Bc*AlyPL6 (22) is a monomer, whereas two other two-domain PL6 enzymes, AlyGC and Patl3640, are dimers (25, 64).

**Figure 4:**
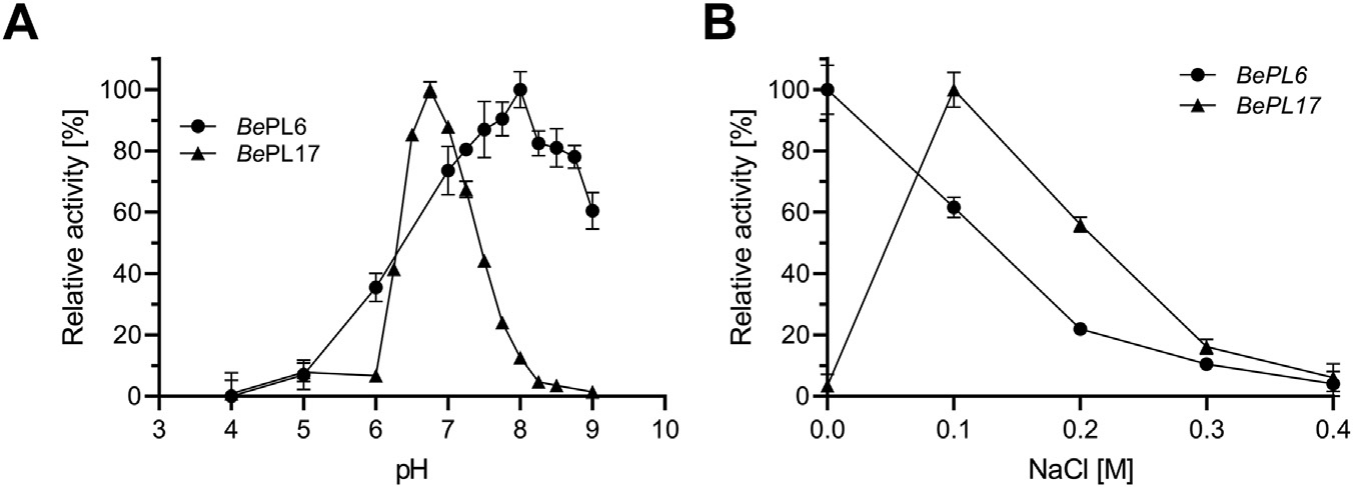
**pH and NaCl dependence of *Be*PL6 and *Be*PL17 activity**. Relative activity of 200 nM *Be*PL6 and 100 nM *Be*PL17 towards 2 mg mL^–1^ alginate at 37 °C. *A*, pH 4.0−9.0 in 20 mM UB4 buffer (90) and *B*, 0.0−0.4 M NaCl in 20 mM HEPES at pH 8.0 and 6.8 for *Be*PL6 (*circle*) and *Be*PL17 (*triangle*), respectively. Samples are run in triplicate and presented as the mean ± SD.

*Be*PL6 has a broad activity optimum towards alginate at pH 7−9 with maximum at pH 8.0 (Fig. 4*A*) and requires no presence of NaCl (Fig. 4*B*). The activity was lost completely at pH 4 and only 4% remained in 0.4 M NaCl (Fig. 4*B*). *Be*PL17 has a narrower pH-activity profile with a maximum at pH 6.8 (Figs. 4*A*) and 0.1 M NaCl (Fig. 4*B*). It was not active at pH 4.0 and only 8% activity remained at pH 5−6 (Fig. 4*A*). Without NaCl and in 0.4 M NaCl 3% and 6% activity was retained, respectively (Fig. 4*B*). *Be*PL6 and *Be*PL17 have melting temperatures (*T*_m_) of 48.5 °C and 51.0 °C, respectively (Fig. S3).

**Figure 5:**
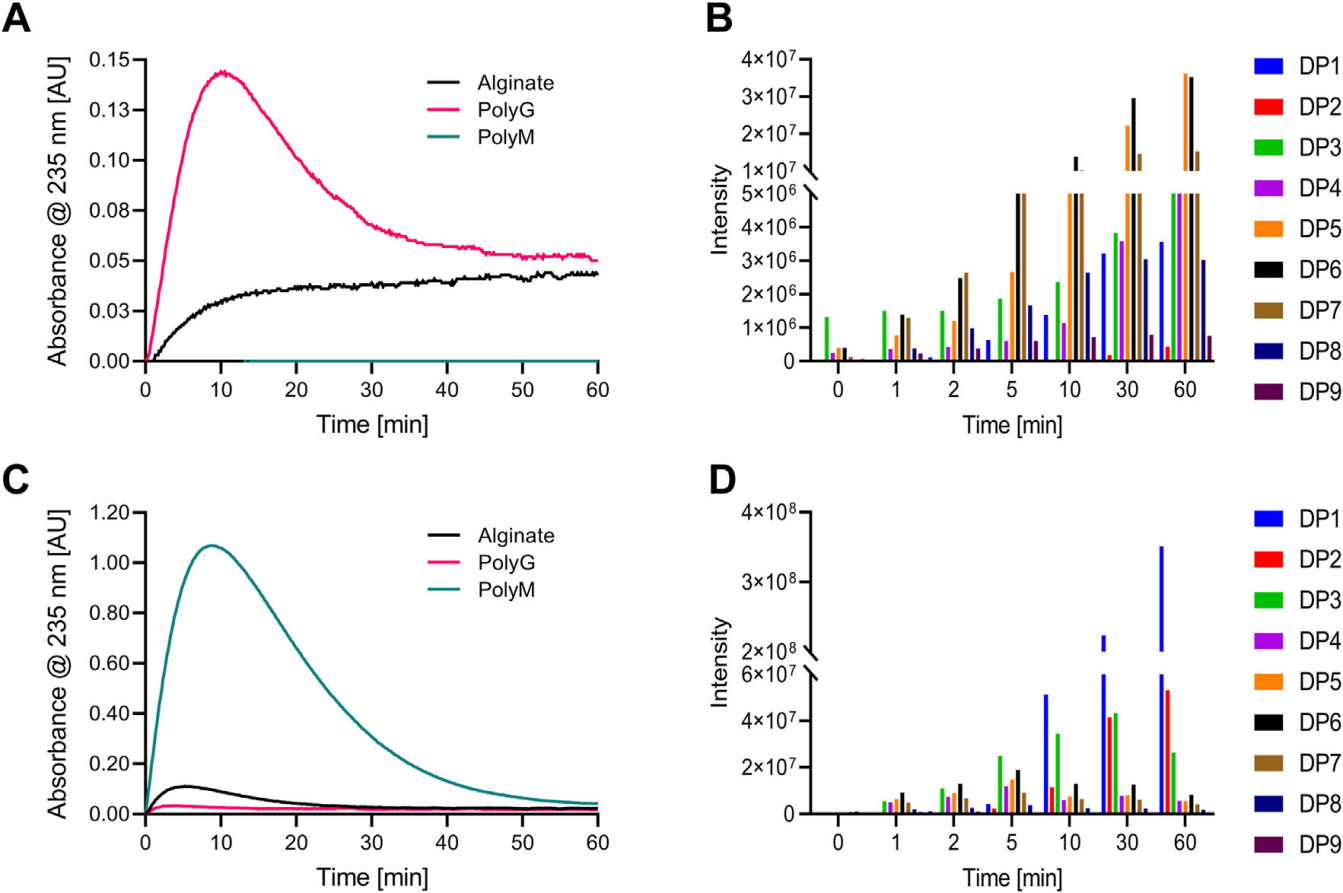
**Specificity and mode of action of *Be*PL6 and *Be*PL17**. *A*, progress of absorbance at 235 nm from 0.38 mg mL^−1^ alginate, polyG and polyM incubated with 100 nM *Be*PL6. *B*, product formation by 100 nM *Be*PL6 from 5 mg mL^−1^ polyG determined by LC-ESI-MS. *C*, progress of absorbance at 235 nm from 0.38 mg mL^−1^ alginate, polyG and polyM incubated with 100 nM *Be*PL17. *D*, product formation by 100 nM *Be*PL17 from 5 mg mL^−1^ polyM determined by LC-ESI-MS. Assay performed in 50 mM HEPES, 150 mM NaCl, pH 7.7. Samples are run as a single sample with no technical replications.

*Be*PL6 catalyzed the degradation of alginate and polyG with a clear preference for polyG and was not active on polyM (Fig. 5*A*). The reaction followed Michaelis-Menten kinetics (Fig. S4) with *k*_cat_ = 12.3 ± 0.1 s^–1^ on polyG being 5-fold higher than for alginate, while the *K*_m_ values of 2.2 ± 0.05 mg mL^−1^ on polyG and 2.8 ± 0.2 mg mL^‒1^ on alginate were very similar (Table 2). *Be*PL17 degraded alginate, polyG and polyM according to Michaelis-Menten kinetics (Fig. S4) with a preference for polyM having *k*_cat_ = 50.9 ± 0.8 s^‒1^, *K*_m_ = 1.1 ± 0.07 mg mL^‒1^ and catalytic efficiency (*k*_cat_/*K*_m_) 8- and 17-fold higher than on alginate and polyG, respectively (Table 2). The observed low activity for polyG likely stems from the reaction on the 7% M residues present in the substrate, rather than the degradation of the polyG part. The time progress of *Be*PL6 degrading polyG first showed an increase in absorbance at 235 nm, which monitors the formation of 4,5-unsaturated monouronates products, followed by a decrease from about 10 min (Fig. 5*A*). This loss in absorbance at 235 nm arises from spontaneous ring opening and contraction of released Δ, of which the formation was confirmed as DP1 by LC-ESI-MS (Fig. 5*B*). After about 50 min the absorbance at 235 nm (Fig. 5*A*) was retained for at least 4 h (data not shown). This behavior indicates that both Δ and unsaturated AOSs are formed, and LC-ESI-MS identified AOSs of DP2‒9, with DP5 and DP6 dominating and not yet declining at 60 min (Fig. 5*B*). As the intensity for DP2‒9 unsaturated AOSs from polyG did not decrease during 1 h of reaction, *Be*PL6 seems not to degrade these AOSs. It is therefore suspected that a small amount of DP1 is produced from oligosaccharides longer than DP9 or polysaccharides. From alginate *Be*PL6 released increasing amounts of DP1 and AOSs of DP2−9 (Fig. S5*A*) indicating degradation of accessible G blocks. *Be*PL6 thus showed some *exo*-action releasing Δ, while the release of AOSs may happen by *exo*- and/or *endo*-action. LC-ESI-MS confirmed the spectrophotometric assay indicating that *Be*PL6 did not degrade polyM (data not shown).

**Table 2:** Kinetic parameters for *Be*PL6 and *Be*PL17 on alginate, polyM and polyG

Enzyme	Substrate	*k*_*cat*_ [s^‒1^]	*K*_m_ [mg mL^‒1^]	*k*_*cat*_*/K*_m_ [mL s^‒1^ mg^‒1^]
*Be*PL6	Alginate	2.6 ± 0.1	2.8 ± 0.2	0.9
	PolyM	n.d.	n.d.	n.d.
	PolyG	12.3 ± 0.1	2.2 ± 0.1	5.6
*Be*PL17	Alginate	27.0 ± 0.3	4.6 ± 0.1	5.9
	PolyM	50.9 ± 0.8	1.1 ± 0.1	46.3
	PolyG	34.6 ± 0.8	13.1 ± 0.4	2.6

Michaelis-Menten plots are shown in Fig. S4. n.d., not detected.

*Be*PL17 degraded polyM to Δ as suggested by the initial increase followed by a loss in absorbance at 235 nm from about 10 min assumed to stem from ring opening and contraction of Δ (Fig. 5*C*). The very low absorbance at 235 nm after 60 min indicated that only trace amounts of unsaturated AOSs persisted, as verified by LC-ESI-MS (Fig. 5*D*). A slight increase in unsaturated DP2 was observed, but DP3–9 were consumed. *Be*PL17 thus degrades DP3 and longer AOSs to Δ. Notably, DP2 seemed not to be degraded. The activity of *Be*PL17 was very low towards polyG and alginate. As polyG is prepared by alginate epimerase-catalyzed conversion of polyM (65), residual M residues are found at the end of polyG, which can give rise to the apparent low activity of *Be*PL17 on this substrate. Similarly, low activity towards alginate may stem from the degradation of M-blocks at the polysaccharide ends (Fig. 5*C*). LC-ESI-MS analysis of AOS products thus showed a very slight increase of DP1 from polyG and alginate (Fig. S5).

### Recombinant production and biochemical characterization of the KdgF-like enzyme, *Be*KdgF

Genes encoding KdgF-like enzymes are widely distributed among different phyla in Bacteria and Archaea (29, 41, 43, 55). Thus 3509 sequences (July 5th, 2022) were retrieved from the NCBI clustered database (clustered at 90% using MM2Seq) (66) with an e-value below 10^−5^. *Be*KdgF shares 41.23% and 45.95% sequence identity with *Ha*KdgF and *Ye*KdgF, respectively, the only two previously characterized KdgF-like enzymes (55). This motivated the present recombinant production and characterization of *Be*KdgF, which was obtained in good yield (59 mg L^−1^) and migrated in SDS-PAGE as a single band between 10 and 15 kDa (Fig. S2) in agreement with the theoretical molecular mass of 14,137 Da. SEC-MALS analysis indicated that *Be*KdgF oligomerizes in solution to two species of molecular mass 57.5 ± 0.5 and 26.3 ± 0.3 kDa corresponding to an equilibrium represented by 6.5% tetramer and 93.5% dimer (Fig. S6). Both of these oligomeric states of *Be*KdgF were identified by X-ray crystallography and the presence of a dimer was also confirmed by protein dynamics using NMR spectroscopy (see below).

### Mode of action and kinetics for *Be*KdgF

**Figure 6:**
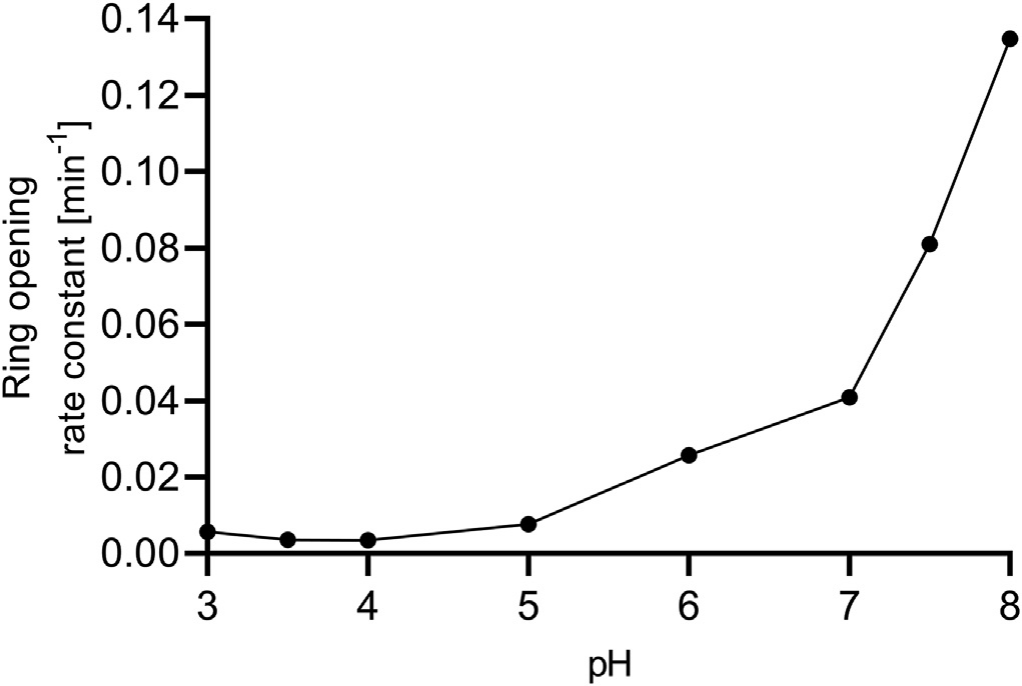
**The pH dependence of the rate of spontaneous conversion of Δ.** Ring-opening, tautomerization, hydration, and contraction of Δ (see Fig. 7) slow down below pH 5 and increase importantly at pH > 7. Samples are run in triplicate and presented as the mean ± SD (for several measurements error bars and mean overlap).

**Figure 7:**
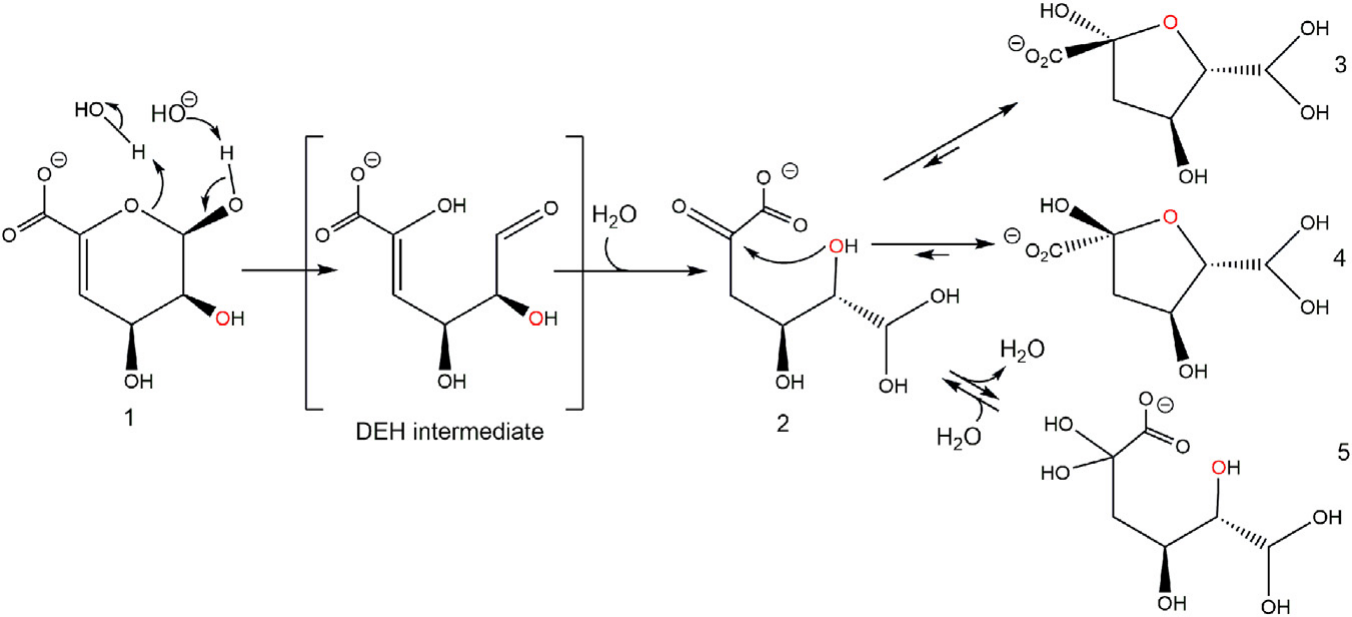
**Base-catalyzed ring opening and contraction of Δ.** The reaction scheme shows the expected reactions spontaneously occurring of Δ (compound 1). O2 is indicated in red. The displayed structures are consistent with the mechanism proposed for spontaneous linearization, tautomerization, hydration, and condensation as monitored by NMR spectroscopy and described in detail by Arntzen *et al.* (8) and NMR spectra presented by Hobbs *et al.* (55). The DEH intermediate is short-lived and not detected by NMR but hydrated to form a ketoaldo-hydrate (compound 5) and an aldo-hydrate (compound 2) of DEH (4-deoxy-L-*erythro*-5-hexoseulose uronate), which undergoes condensation to compounds 3 and 4 (8).

The 4,5-unsaturated uronate, Δ, released by *Be*PL6 and *Be*PL17 acting on polyG, polyM, and alginate (Fig. 5, *A* and *C*) undergoes spontaneous ring opening, which appears to be base-catalyzed as evidenced by the prominent increase in the reaction rate at pH > 5 (Fig. 6). The ring opening of Δ (compound 1, Fig. 7) to DEH is thus akin to base-catalyzed mutarotation (67). According to the literature (8), the DEH intermediate is short-lived and not detected by NMR and immediately undergoes enol-keto tautomerization and hydration to DEH aldo-hydrate (compound 2) and ketoaldo-hydrate (compound 5) (8). The order in which tautomerization and hydrations occur could not be monitored, as only the product of both these reactions was observed (compound 2), as well as compounds 3, 4, and 5, with which compound 2 is in equilibrium. Compounds 3 and 4 are predominant and formed by cyclization (contraction) by OH-2 attacking the 5-keto group of compound 2, leading to the formation of two furanosides, (5*S*)-DHF hydrate and (5*R*)-DHF hydrate (compounds 3 and 4) (Fig. 7). Note, that in this spontaneous reaction, the first step is rate-limiting (8), hence the loss of absorbance at 235 nm can be directly linked to the conversion of compound 1. Moreover, note that under acidic conditions, another mechanism takes place leading to the formation of the desaturated 5-formyl-2-furoic acid (68).

**Figure 8:**
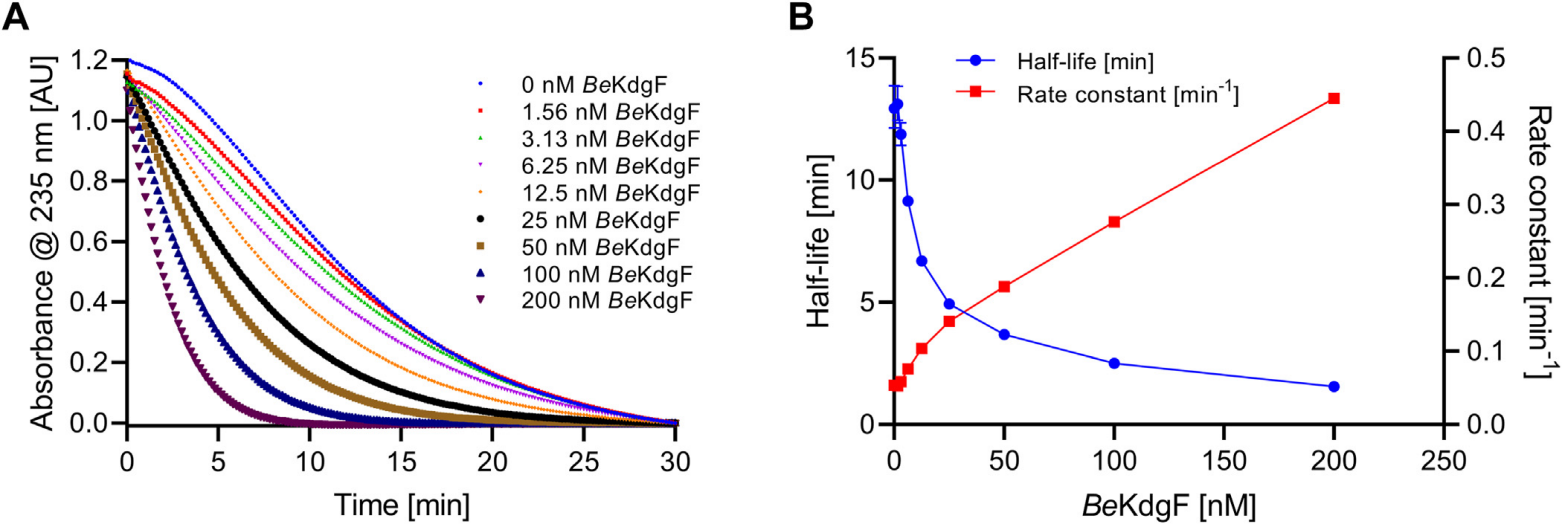
***Be*KdgF catalyzed conversion of Δ.***A*, from 0 to 200 nM *Be*KdgF was directly added to the substrate Δ of an estimated concentration of 250 μM in 50 mM HEPES, 150 mM NaCl, pH 7.7 formed from 0.5 mg mL^−1^ polyM by 100 nM *Be*PL17 (at the time point where the change in absorbance at 235 nm reached a plateau). *B*, Half-life and rate constant calculated by fitting a one-phase decay model to the data obtained in *A*.

The rate of spontaneous conversion of Δ was <0.005 min^‒1^ at pH 4, and 0.13 min^‒1^ at pH 8 (Fig. 6). Hence, adjusting to pH 3 after the release of Δ from polyM by *Be*PL17 ensured a known Δ concentration due to the reduced spontaneous conversion of Δ. Using this Δ as substrate, the rate of conversion of approximately 250 μM Δ at pH 7.7 catalyzed by 0−200 nM *Be*KdgF increased in a dose-dependent manner (Fig. 8*A*). By comparing half-lives and rate constants of the absorbance decrease at 235 nm, *Be*KdgF is observed to catalyze a reaction accompanied by loss of absorbance at 235 nm, here associated with ring opening and tautomerization to compound 2 (Fig. 7). The enzyme-catalyzed reaction occurred by 8-fold higher rate (Fig. 8*B*) than the corresponding spontaneous reaction, of which the rate was determined to 0.05 min^–1^ as displayed at [*Be*KdgF] = 0 (Fig. 8*B*). Activities of *Be*KdgF were subsequently determined as the slope of the rate as a function of [*Be*KdgF], which accounts for the contribution of the background reaction due to the spontaneous conversion of Δ.

**Figure 9:**
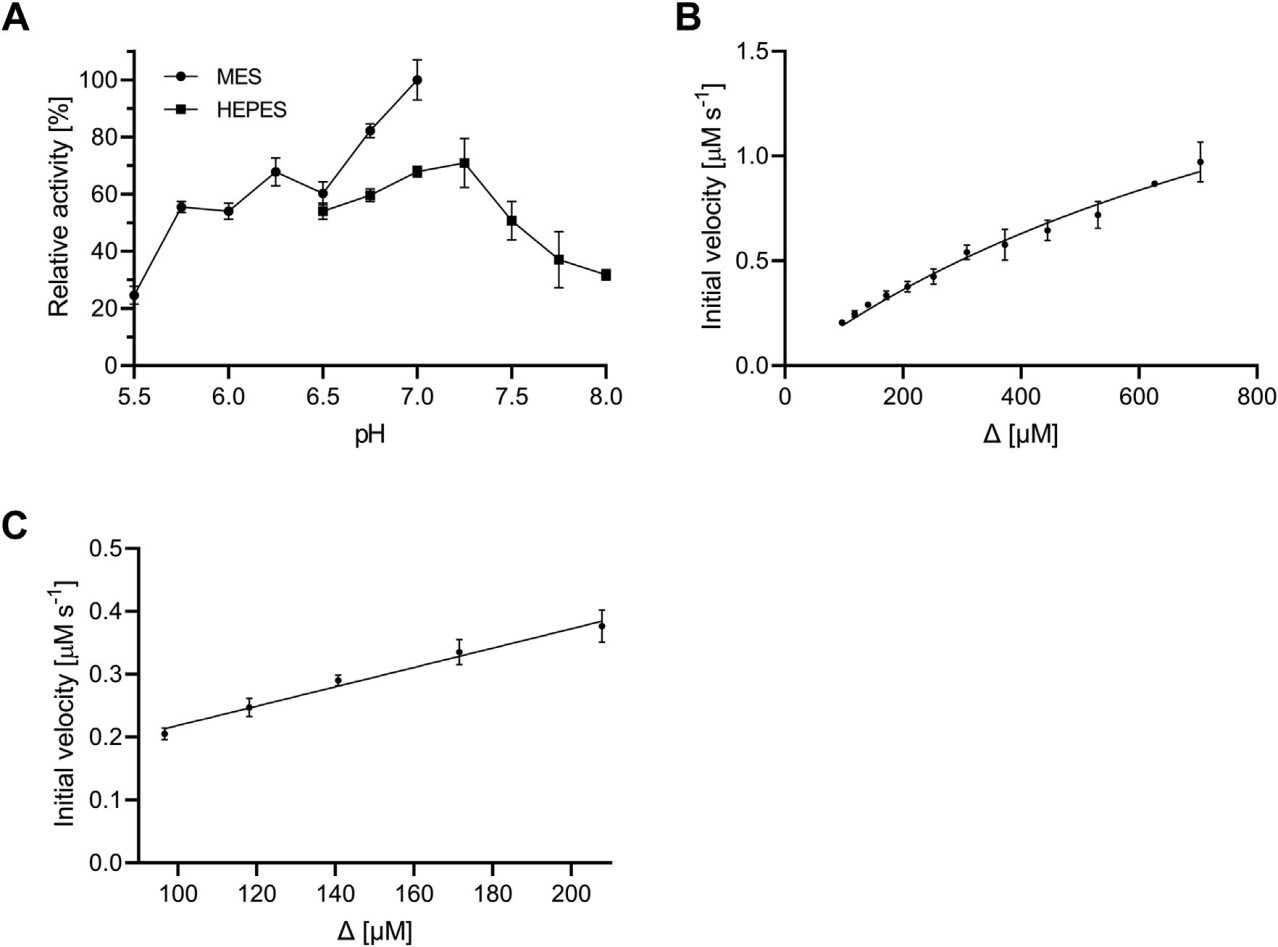
***Be*KdgF pH optimum and kinetics.***A*, the specific activity was determined at three enzyme concentrations (0, 10 and 30 nM *Be*KdgF) as described in Experimental procedures, towards 250 μM Δ at pH 5.5 to 7.0 in 50 mM MES, 150 mM NaCl (*circle*) and at pH 6.5 to 8.0 in 50 mM HEPES, 150 mM NaCl (*square*). *B*, Michaelis-Menten plot for 20 nM *Be*KdgF on Δ in 50 mM HEPES, 150 mM NaCl, pH 7.0. *C*, linear fit of reaction rate vs low Δ concentrations yielding *k*_cat_/*K*_m_ = 0.08 μM^‒1^ s^‒1^. Samples are run in triplicate and presented as the mean ± SD.

*Be*KdgF appears to have a fairly broad pH activity dependence with a maximum of around pH 7 (Fig. 9*A*). Both broad activity optima and varying effects of different buffer components, similar to what was found here for *Be*KdgF (Fig. 9*A*), have been reported for other enzymes of the cupin superfamily (69, 70).

In the case of *Be*KdgF even though the lack of robust stability of Δ under relevant assay conditions did not allow a very accurate analysis, our control of the availability of Δ by lowering pH, made it possible to estimate kinetic parameters (Fig. 9*B*). Although substrate saturation of *Be*KdgF was not achieved the parameters *k*_cat_ = 121.2 ± 42.1 s^‒1^, *K*_m_ = 1.14 ± 0.56 mM, and *k*_cat_/*K*_m_ = 0.11 μM^‒1^ s^‒1^ were obtained by fitting the experimental data (R^2^ = 0.96) to the Michaelis-Menten equation, which is in good agreement with *k*_cat_/*K*_m_ = 0.08 μM^‒1^ s^‒1^ determined from the linear relationship at low substrate concentration (Fig. 9*C*).

### Metal dependence of *Be*KdgF

**Figure 10:**
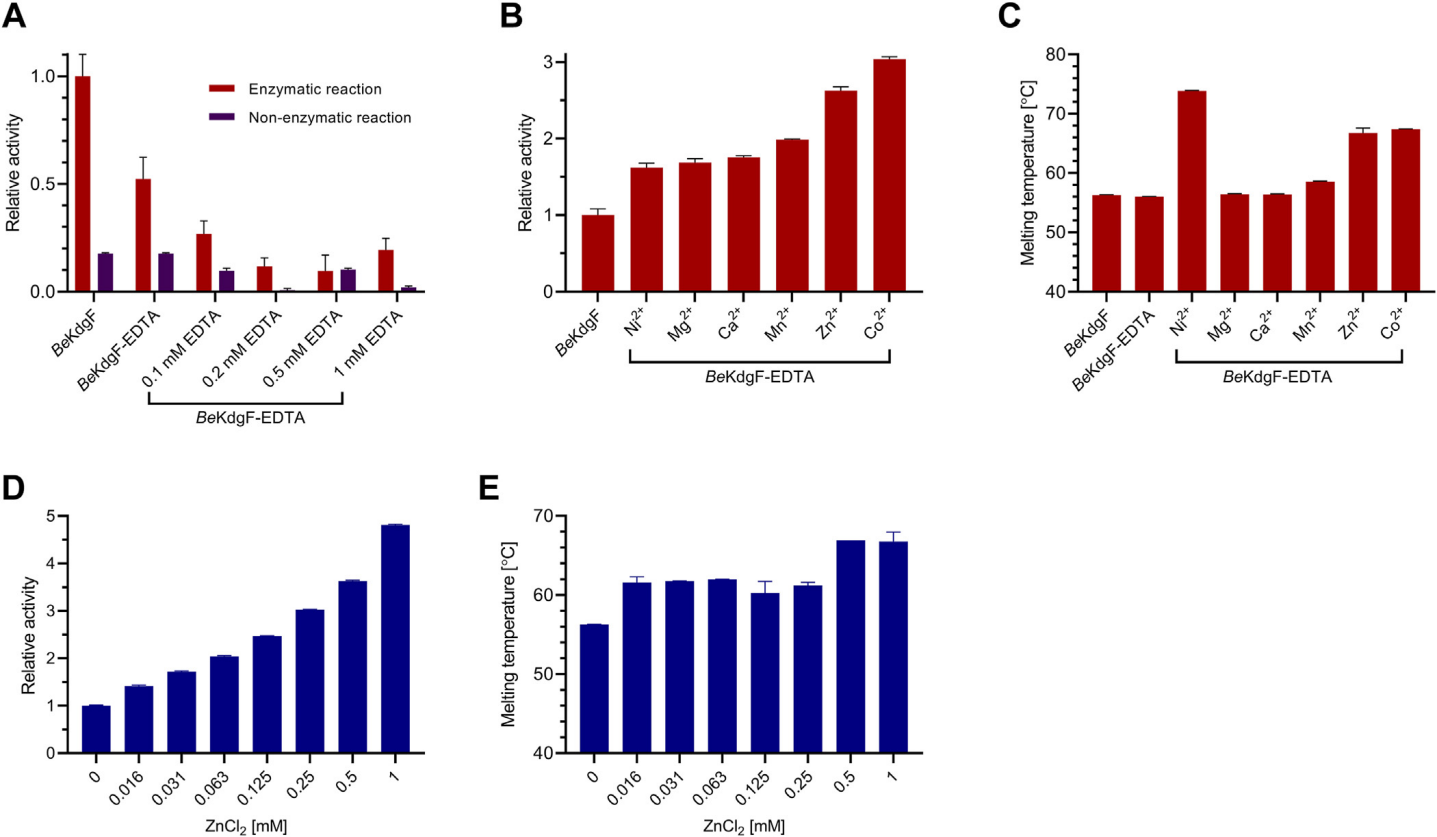
**Activity and stability of *Be*KdgF, EDTA-treated and added divalent metal ions.***A*, background-corrected activity of 100 nM *Be*KdgF on 498 μM Δ before (*Be*KdgF), after pre-treatment by 50 mM EDTA (*Be*KdgF-EDTA) and subsequent addition of 0.1−1 mM EDTA in the assay mixture (*red*). Non-enzymatic reaction of 498 μM Δ with and without the addition of 0.1−1 mM EDTA (*blue*). *B*, the activity of 100 nM *Be*KdgF on 348 μM Δ before and after pre-treatment by 50 mM EDTA (*Be*KdgF-EDTA) and subsequent addition of 1 mM divalent metal ions. C, *T*_m_ of 130 μM *Be*KdgF and *Be*KdgF-EDTA without and after the addition of 1 mM divalent metal ions. *D*, activity at 0−1 mM Zn^2+^ (normalized for *Be*KdgF-EDTA). *E*, *T*_m_ of *Be*KdgF at 0−1 mM Zn^2+^ (calculated from Fig. S7). All assays were performed in 20 mM HEPES, 150 mM NaCl, pH 7.0. Samples are run in triplicate and presented as the mean ± SD.

Pre-treatment of *Be*KdgF with 50 mM EDTA (*Be*KdgF-EDTA) caused approximately 50% reduction in activity towards Δ (Fig. 10*A*), different from the pectinolytic *Ye*KdgF reported to lose all activity after treatment with EDTA (55). Notably, the addition of 0.1−1 mM EDTA to the *Be*KdgF assay mixture decreased activity to 10−25% of that of the recombinant enzyme (Fig. 10*A*). It remains unknown if the loss of activity stemmed solely from *in situ* elimination of cations, or if *Be*KdgF was also inhibited due to EDTA binding to the active site. Moreover, in the presence of 0.1−1 mM EDTA, the rate of the spontaneous conversion of Δ was suppressed by 50−95% (Fig. 10*A*). While Zn^2+^ and Co^2+^ increased the activity of EDTA pre-treated *Be*KdgF by 2.6 respectively 3.0-fold, all other tested metals increased activity by 1.5−1.9-fold (Fig. 10*B*). Metal ions similarly although slightly differently increased the activity of *Ye*KdgF and *Ha*KdgF (55). Notably, *Be*KdgF showed good stability, and after EDTA-treatment addition of Ni^2+^, Co^2+^ or Zn^2+^ raised *T*_m_ very importantly from 56.2 to 73.9, 67.4, and 66.8 °C, respectively, the increase by Ni^2+^ thus amounting to 17.6 °C (Fig. 10*C*). Although Zn^2+^ and Co^2+^ stimulated *Be*KdgF activity the most (Fig. 10*B*), titration with Zn^2+^ was investigated for *Be*KdgF as zinc is more biologically relevant and was found in similar proteins, such as different enzymes of the cupin family (71, 72, 73). Remarkably, increasing Zn^2+^ up to 1 mM resulted in 5-fold higher activity of *Be*KdgF-EDTA (Fig. 10*D*).

We think that the interaction of EDTA is complex and the data represent a combined effect of (i) EDTA binding at the active site, similar to *e.g.* malonate, citrate, or glycerol found in the three different KdgF crystal structures of *Ye*KdgF, *Ha*KdgF, and *Be*KdgF, and that EDTA in this way acts as an inhibitor, (ii) removal of metal by EDTA chelation, and (iii) EDTA suppressing the spontaneous conversion of Δ. We did not pursue unraveling these individual effects due to the inherent complications associated with substrate production, stability, and enzyme activity assaying.

### X-ray crystallographic analysis of *Be*KdgF

**Figure 11:**
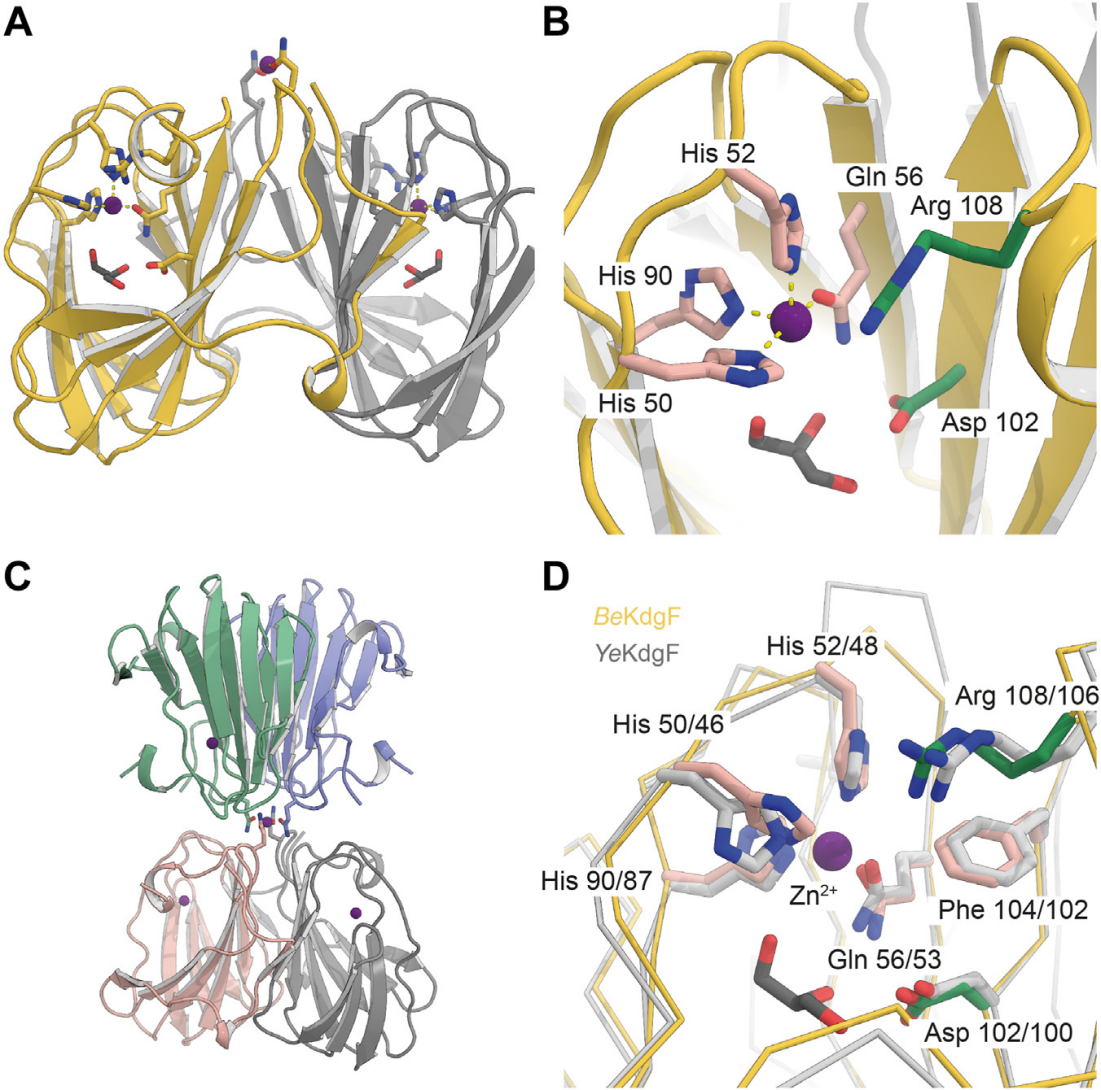
***Be*KdgF crystal structure**. *A*, overall structure of the *Be*KdgF-Zn dimer with the glycerol molecule and the metal binding residues highlighted. *B*, active site residues (*green* and *salmon sticks*). *C*, organization of the metal-induced tetramer. *D*, comparison of conserved catalytically important residues required for activity in *Ye*KdgF (*grey*) and *Be*KdgF (*salmon*), and conserved metal coordinating residues (*Be*KdgF His50, His52, Glu56, and His90. Glycerol is shown in *solid black sticks*.

Screening of crystallization conditions yielded well-diffracting crystals and data sets of 1.5 Å and 2.0 Å resolution for recombinant *Be*KdgF (*Be*KdgF-Ca) (PDB: 7ZYB) and EDTA-depleted *Be*KdgF reconstituted by addition of ZnCl_2_ (*Be*KdgF-Zn) (PDB: 7ZYC), respectively. The structures were solved by molecular replacement using one monomer from the closest homolog, *Ye*KdgF (PDB: 5FPX, 46% sequence identity to *Be*KdgF) as a search model leading to two similar models of *Be*KdgF-Ca with a *R*_free_ of 20.08% (Table S2) and *Be*KdgF-Zn having *R*_free_ of 25.97%. Both structures have one protomer in the asymmetric unit with a Matthews coefficient of 1.89 for *Be*KdgF-Ca and 1.84 for *Be*KdgF-Zn. These structures exhibit the canonical β-barrel cupin fold (74) and closely resemble the structures of *Ye*KdgF and *Ha*KdgF (55). The dimeric assembly of *Be*KdgF (Fig. 11*A*) predicted by the PISA server (75) has the same homodimeric interface as *Ye*KdgF and *Ha*KdgF (PDB: 5FQ0) (55). At the active site of *Be*KdgF, electron density modeled well a glycerol molecule (Fig. 11, *A* and *B*), that sits slightly deeper into the active site than the malonate molecule observed in *Ye*KdgF (55). Residues in *Ye*KdgF (Asp100, Phe102, and Arg106) proposed as important for catalysis, are conserved in *Be*KdgF as Asp102, Phe104, and Arg108, and structurally oriented in very similar positions as in the homolog (55) (Fig. 11*D*). In both *Be*KdgF structures, electron density corresponding to a metal ion is observed at approximately the same position where Ni^2+^ is modeled in the active site of *Ye*KdgF (Fig. 11*D*).

The metal binding site of *Be*KdgF was further investigated because biochemical data indicated that its activity did not depend as rigorously on metal ions (Fig. 10*A*) as found for *Ye*KdgF (55). In the *Be*KdgF structure, the metal ion is modeled as Ca^2+^, which is present in the crystallization conditions and is consistent with suitable refinement statistics, whereas for the structure of *Be*KdgF-Zn the metal is modeled as a Zn^2+^ also revealing a more defined electron density. Refinement to optimize occupancy gave 50 and 80% occupancy for Ca^2+^ and Zn^2+^, respectively, whereas Ni^2+^ in the homologous *Ye*KdgF showed 100% occupancy. The residues coordinating the metal are conserved and comprise three histidines and one glutamine, forming an octahedral binding geometry compatible with the assayed divalent cations (Fig. 11*D*). This lower metal occupancy for *Be*KdgF may be a distinctive characteristic reflected also by the biochemical data obtained for *Be*KdgF indicating the slightly less critical metal influence on activity, as opposed to the two homologs, *Ye*KdgF and *Ha*KdgF (55), and higher B-factors for metal ligands in *Be*KdgF as compared to in *Ye*KdgF and *Ha*KdgF (Table S3). An additional finding for *Be*KdgF is the presence of electron density indicating a metal ion on the exterior of the structure, coordinated by Glu33, eliciting the formation of a tetramer (a dimer of dimers) (Fig. 11*C*), which was not reported for the two homologs (55).

### Structural analysis of *Be*KdgF in solution by NMR

The structure of *Be*KdgF was investigated in aqueous solution by using NMR spectroscopy. The secondary structure was primarily β-sheets in accordance with the crystal structure (76). A dynamic analysis of *Be*KdgF made by measuring ^15^N *T*_*1*_ and ^15^N *T*_*2*_ relaxation times and {^1^H}-^15^N NOEs of the protein (Fig. S8) showed that the flexibility of the protein is greater at the C and N termini. The rotational correlation time (τ_c_) for *Be*KdgF was calculated based on *T*_1_ and *T*_2_ to τ_c_ = 15.3 ns ± 0.7 ns (77). Globular proteins have a distribution of mass, which gives rotational correlation time τ_c_ approximately 0.6 times their molecular weight (78). For *BeK*dgF this indicates a dimeric state in solution as the tumbling fits a protein of 25 ± 1.2 kDa.

**Figure 12:**
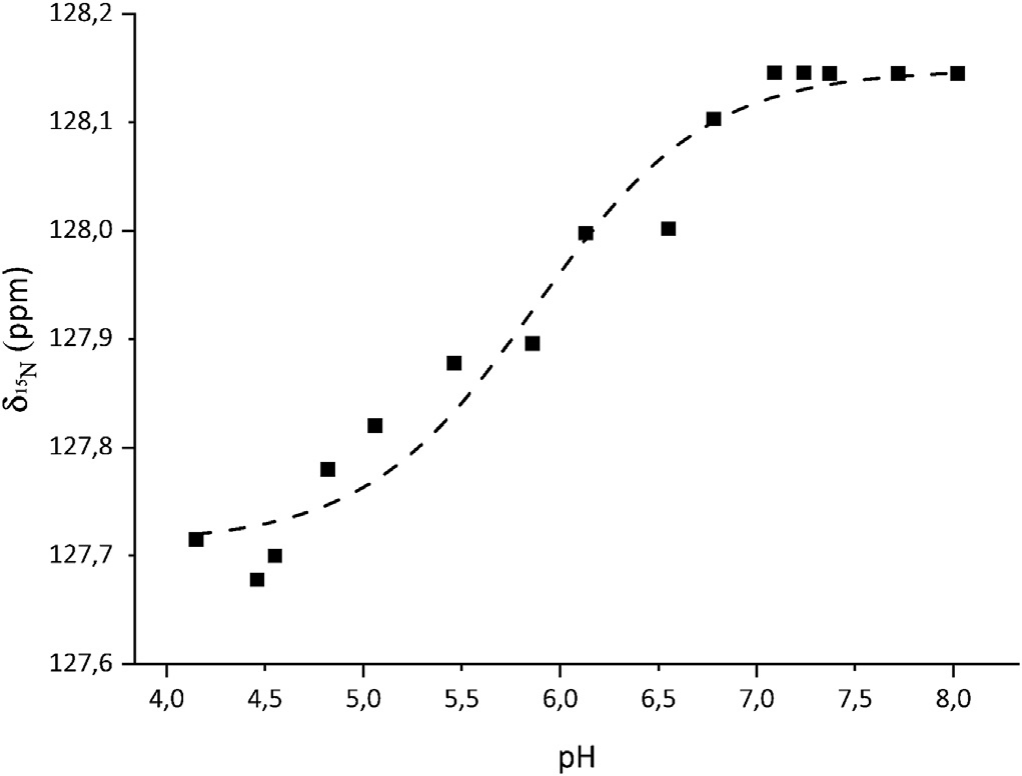
**pH titration of the active site residue Asp102 in *Be*KdgF**. The titration of Asp102 was followed using the N^H^ chemical shift (δ ^15^N) as function of pH. Using the Henderson-Hasselbalch equation, the p*K*_a_ was calculated to 5.9 ± 0.1 (79).

A pH titration was performed in the range pH 4.15−8.02, where a ^15^N HSQC spectrum was recorded at each point of a 15-step titration (Fig. 12). Here the p*K*_a_ of the active site residue Asp102 was determined to 5.9 ± 0.1 by fitting the chemical shift change as a function of pH to the Henderson-Hasselbalch equation (79). This correlated well with Asp102 being proposed as the catalytic base. p*K*_a_ values could also be measured for six other residues titrating in the pH range, but these were not situated at the active site (Table S4 and Fig. S9). It was not possible to perform this titration above pH 8.0 and below pH 4.2.

### Cooperative degradation of alginate by *Be*PL6, *Be*PL17 and *Be*KdgF

**Figure 13:**
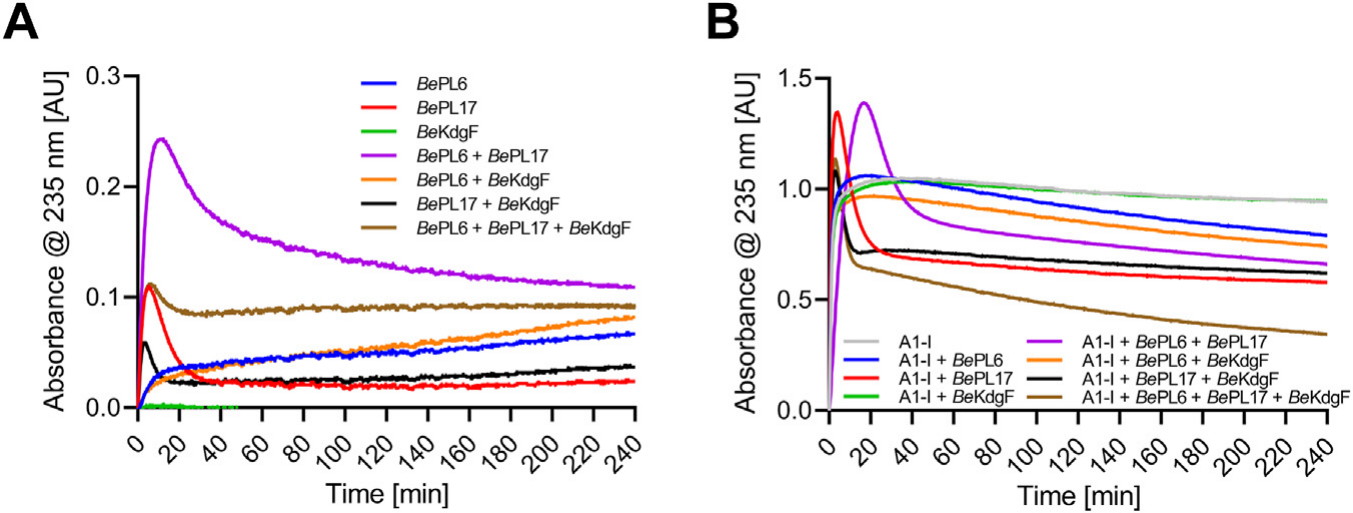
**Cooperative action of *B. eggerthii* alginate degrading enzymes**. Alginate (0.5 mg mL^−1^) in 50 mM HEPES, 150 mM NaCl, pH 7.7 was degraded by 100 nM of each enzyme either alone or in various combinations. *A*, *Be*KdgF, *Be*Pl6, *Be*PL17 and (*B*) the same enzymes as in (*A*) combined with 100 nM A1-I from *Sphingomonas* sp. A1. Samples are run in triplicate presented here as the mean value of the data points.

Regarding alginate utilization by *B. eggerthii* the cooperation of *Be*PL6, *Be*PL17, and *Be*KdgF was investigated for 100 nM of each of the three enzymes (Fig. 13*A*). Moreover, to relate to the applied culture condition mimicking cross-feeding, the effect was also determined of the addition of 100 nM A1-I *endo*-acting alginate lyase from *Sphingomonas* sp. A1, which produced AOSs of predominantly DP2−4 (Figs. 13*B* and S1). The reaction on 0.5 mg mL^-1^ alginate in 50 mM HEPES, 150 mM NaCl, pH 7.7 was monitored by the change in absorbance at 235 nm for the single enzymes and various combinations thereof. *Be*PL6 alone caused a small increase followed by a slow decline in absorbance at 235 nm, while *Be*PL17 showed higher initial increase followed by a rapid decline. As expected *Be*KdgF alone had no activity on alginate. The two lyases combined resulted in the highest and synergistic degradation, as this exceeded the additive effect of the *Be*PL6 and *Be*PL17 acting individually (Fig. 13*A*). Notably, combining each of the two lyases with *Be*KdgF considerably reduced the initial absorbance at 235 nm generated by *Be*PL17 alone, while the combination of *Be*KdgF and *Be*PL6 hardly differed from the effect of *Be*PL6 alone (Fig. 13*A*) reflecting that *Be*PL6 forms AOSs rather than Δ, the 4,5-unsaturated monouronate, which was generated by *Be*PL17 and is the substrate for *Be*KdgF. This and the reaction of all three enzymes combined indicated a shallow plateau to persist as also obtained with *Be*PL6 alone, thus the lyase products were not all converted to Δ. The addition of the very efficient *endo*-acting lyase A1-I resulted in much higher product yields from alginate (Fig. 13*B*), and moreover, the absorbance at 235 nm still decreased after 4 h when all four enzymes were present indicating continued degradation with some release and subsequent ring opening and condensation of Δ. The *exo*-action by *Be*PL17 to produce Δ was evident from the fast increase followed by a fast decrease of absorbance at 235 nm. When *Be*PL6 and A1-I were present, in the later phase a slow albeit slightly faster loss of absorbance at 235 nm, hence ring opening and condensation of Δ, resulted compared to reactions by *Be*PL17 and A1-I, without *Be*PL6 (Fig. 13*B*). This may reflect a relatively poor ability of *Be*PL17 to degrade some of the AOSs formed by A1-I.

## Discussion

Although the two alginate lyases *Be*PL6 and *Be*PL17 are capable of degrading alginate, since *B. eggerthii* does not encode an extracellular *endo*-acting alginate lyase it depends on AOSs released by other bacteria from alginate, a cooperative action of HGM members referred to as cross-feeding (80, 81, 82). It is well-documented that microbial communities represent an intricate network of different interactions. However, investigation on potential cross-feeding between *B. eggerthii* and other HGM members in alginate degradation is beyond the scope of the present study. The SusC_H_/SusD_H_ system binds and transports oligosaccharides rather than polysaccharides in accordance with previous reports (60). *Be*PL6 and *Be*PL17, both containing signal peptides, are most probably located in the periplasmic space and the lack of an extracellular alginate lyase in *B. eggerthii* is thought to be the reason for its inability to grow on alginate. The PUL of *B. ovatus* CP926 encodes two more proteins than the PUL of *B. eggerthii*, here most importantly the multi-specific alginate lyase, *Bo*PL38, which seems to be the reason for *B. ovatus* CP926 being able to grow on alginate as sole carbon source, unlike *B. eggerthii*. Notably, the gut microbe *B. thetaiotaomicron* that can utilize both amylopectin and levan was rendered unable to grow on these polysaccharides after the deletion of genes encoding outer surface glycoside hydrolases (83). The same study showed that this is not the case for all organisms, as deleting two outer surface glycoside hydrolases from the inulin utilization locus in *B. ovatus* did not cause loss of growth on inulin. Remarkably, this deletion in *B. ovatus* reduced the cross-feeding ability of *Bacteroides vulgatus* which grows on inulin breakdown products but cannot utilize inulin (83). Cooperative degradation of alginate by *Be*PL6, *Be*PL17, and *Be*KdgF is apparent in accordance with the respective substrate specificities for the two alginate lyases able to release Δ. *Be*PL6 produced AOSs, whereas *Be*PL17 seemed to be only acting in *exo*-mode with release of Δ. Addition of the very efficient *endo*-acting A1-I (46, 57) as expected importantly enhanced the efficiency of alginate degradation. Thus, while *Be*PL6, *Be*PL17, and *Be*KdgF together degraded alginate quite poorly, the supply of AOSs produced by the recombinant A1-I alginate lyase from the marine bacterium *Sphingomonas* sp. strain A1 ensured alginate utilization, possibly emulating cross-feeding in the gut. Notably, addition of recombinant *Be*PL6 and *Be*PL17 to a *B. eggerthii* DSM 20697 culture did not result in growth, probably because these enzymes have about 25-, respectively 10-fold lower activity on alginate than the A1-I (see Fig. 13). Moreover, the AOSs of DP2−4 released from alginate by A1-I may be more readily utilized by *B. eggerthii* DSM 20697 than the AOSs of DP5−7 formed by *Be*PL6 (see Figs. S1 and S5*A*). Δ was still formed after 4 h by enzyme combinations comprising A1-I, and most strongly when all three enzymes *Be*PL6, *Be*PL17 and *Be*KdgF were present, suggesting a continuous and potentially complete degradation of alginate occurred. This was previously observed for enzymes from marine bacteria by mixing the *endo*-acting enzyme AlySY08 from *Vibrio* sp. SY08 (84) with two *exo*-acting enzymes (OalC6 and OalC17) from *Cellulophaga* sp. SY116, improving degradation of alginate to monosaccharides up to 84% (85).

According to the PULDB (37) 180 PULs have the combination of PL6 and PL17 together with as well as without other PLs (23), indicating quite a widespread combination seen in other organisms. This included a shorter version of the PUL in *B. eggerthii*, spanning 6 rather than 10 genes found in the PUL in the present work; both PUL versions encoded a PL6 and a PL17 enzyme (19). However, it is not known how many of the enzymes in these PULs in the PULDB contribute to the breakdown and utilization of alginate. Thus, despite the relatively common co-occurrence of PL6 and PL17 (23), their cooperation has not been studied in detail. However, it has been reported that a combination of a polyG active PL6 and a polyM active PL17 from the marine bacterium *Cellulophaga* sp. *SY116* increased activity on alginate to 150% compared to the sum of the activities of the two individual enzymes (85). While it was shown that *Cellulophaga* sp. *SY116* acts selectively towards alginate, neither growth data nor the presence of other predicted alginate lyases in the genome were reported in that work. *Cellulophaga* sp. *SY116* having only a PL6 and a PL17 enzyme thus seemed capable of utilizing alginate. Moreover, an *endo*- and an *exo*-acting alginate lyase of families PL8 and PL7 from the marine fungus *Paradrendryphiela salina* act synergistically enhancing alginate degradation (86).

Alginate lyases of family PL17 are generally known as *exo*-acting with a strong preference for M-blocks (61). In fact, in a phylogenetic mapping and screening of family PL17 enzymes, *B. eggerthii* PL17 was reported to be *exo*-acting and M-specific, but not characterized in more detail (19). The PL6 family by contrast covers several modes of action and specificities, including towards alginate and chondroitin sulfate (27, 87). Most PL6 members act in *endo*-mode and prefer G-blocks, although M-block specificity was reported for *Bcel*PL6 from the gut bacterium *B. cellulosilyticus* (20, 27, 64). The *Be*PL6 having *k*_cat_ of 12.3 ± 0.1 s^–1^ shows moderate to low activity on polyG, its preferred substrate, compared to other polyG-active PL6 enzymes, *e.g.* OalS6 from *Shewanella* sp. *Kz7* (88) having *k*_cat_ of 38 s^−1^ and OalC6 from *Cellulophaga* sp. SY116 with *k*_cat_ of 109 s^−1^ (85). In addition, even though *Be*PL17 showed high activity on its preferred substrate polyM with a *k*_cat_ of 50.9 ± 0.8 s^−1^, other polyM-active PL17 enzymes have 2.5- and 1.8-fold higher activity (26, 85).

Only two KdgF enzymes have been characterized before, namely *Ye*KdgF from the pectinolytic locus of *Y. enterocolitica* and *Ha*KdgF from the alginolytic locus in a *Halomonas* sp., acting at comparable rates on 4,5-unsaturated monouronate from alginate (55). *Be*KdgF has not been tested on 4,5-unsaturated galacturonate but possesses 45.95% and 41.23% sequence identity and shares very similar structural features with *Ye*KdgF and *Ha*KdgF, respectively. However, while the activity of *Ye*KdgF was shown to depend strictly on metal ions, the activity of *Be*KdgF was not completely eliminated by pre-treatment with 50 mM EDTA or even if 0−1 mM EDTA was present in the assay mixture. Notably, similarly to *Ye*KdgF, where the addition of metal ions increased activity up to 3-fold (55), the addition of metal ions increased activity of *Be*KdgF up to 2.8-fold the level of the native enzyme. Due to the high rate of spontaneous conversion of 4,5-unsaturated monouronate Hobbs *et al.* (55) assayed activity on the substrate generated *in situ* by specific lyases from dimannuronate and digalacturonate and not directly on the pure 4,5-unsaturated monouronate. To overcome this shortcoming, we adjusted *Be*PL17 generated Δ to pH 3 suppressing spontaneous conversion and enabling the first estimation of kinetic parameters for a KdgF enzyme, which can be useful for future KdgF characterization.

### Proposed mechanism of *Be*KdgF

There are still a few unknowns of the mechanism of *Be*KdgF, regarding the possible involvement of metal ions, which steps are actually catalyzed by the enzyme and which are spontaneous, identification of catalytic residue(s), and the detailed molecular mechanism. Metal ions may play a role in *Be*KdgF activity, although EDTA pre-treatment did not fully inactivate *Be*KdgF, and neither did the presence of EDTA during the assay. Notably, it was not possible to distinguish effects arising from removal by EDTA of the metal ion bound to *Be*KdgF from the effect of EDTA interacting as an inhibitor by binding to the active site, and moreover to take into account that EDTA suppressed the spontaneous conversion of Δ. Different metal ions moderately increased the activity of *Be*KdgF from 1.6 to 3-fold depending on the metal (Fig. 10*B*). Together with the strong effect of metals on *Be*KdgF stability, it may hint that this effect on activity is rather due to an effect on the structure than a direct involvement of the metal in the molecular events. Given the relatively small differences observed for different stimulating metal ions, if there is a direct involvement, it contributes very little to the catalysis.

**Figure 14:**
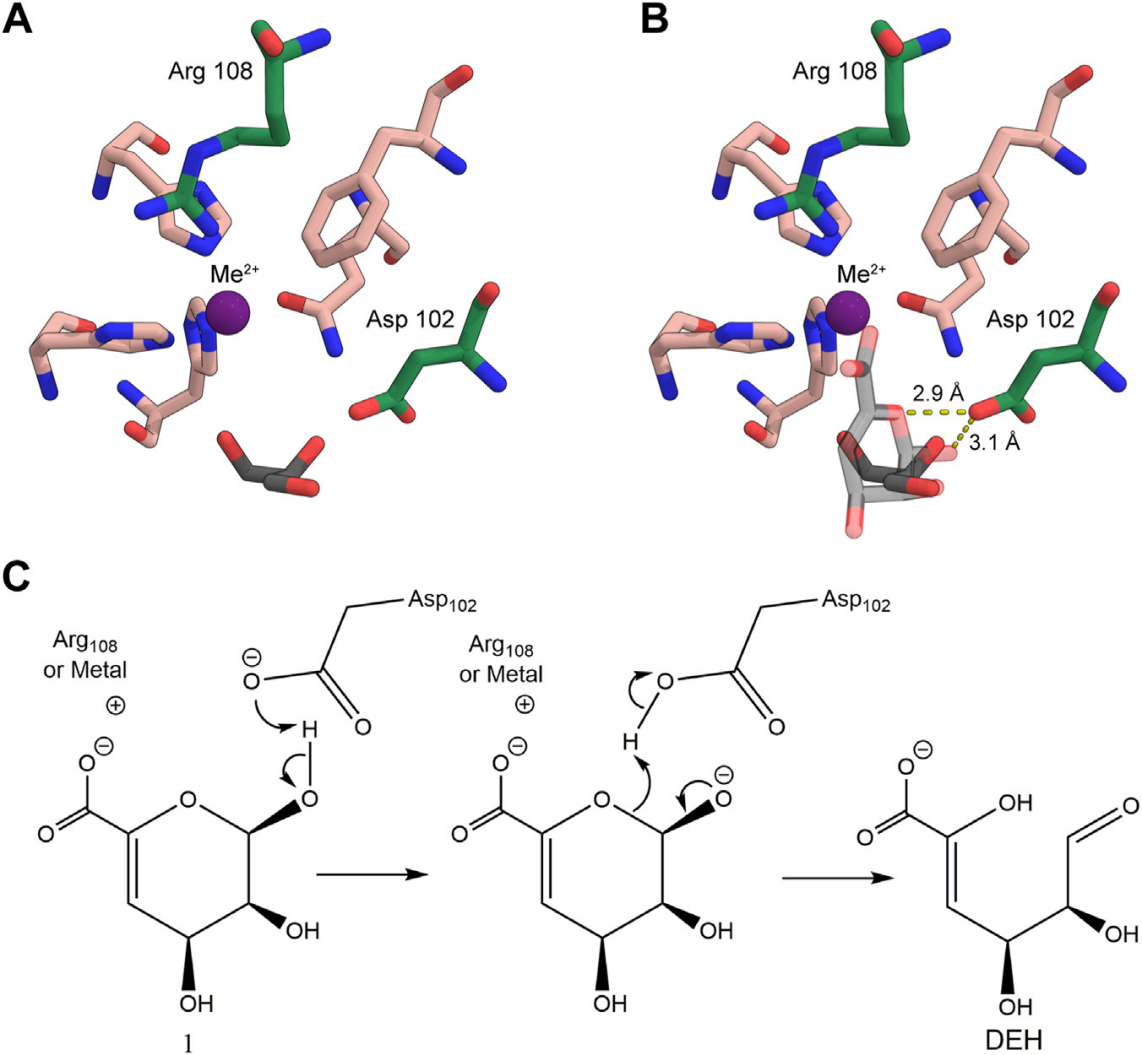
**Proposed mechanism of *Be*KdgF**. *A*, representation of active site with the proposed catalytic acid/base Asp102 along with Arg108, which in combination with the coordinated zinc/metal ion (*purple sphere*) acts by charge neutralization and stabilizes the transition state. The glycerol molecule is shown (*solid black sticks*). *B*, representation of the Δ molecule (transparent *gray sticks*) modeled and refined in the experimental electronic density of the glycerol molecule (*solid black sticks*) in *Be*KdgF. Arg108 and Asp102 are in *green sticks*, other relevant residues are in *salmon sticks*, and the metal ion is a purple sphere. *C*, scheme of the proposed simple mechanism for *Be*KdgF-catalyzed ring opening of Δ yielding DEH, which is tautomerized/hydrated and condensated as shown in Figure 7.

As the loss of absorbance at 235 nm due to the ring opening of Δ depended on the enzyme concentration (Fig. 8*A*), this step was enzyme-catalyzed. The identical ratio between the furanosides (5*S*)-DHF hydrate and (5*R*)-DHF hydrate observed in enzyme-catalyzed and in spontaneous conversion (8, 55), shows that the KdgF enzymes do not catalyze the contraction of DEH hydrate to furanosides (Fig. 7). The question remains whether the enzyme catalyzes only the ring opening, or also catalyzes the subsequent tautomerization, proposed by Hobbs *et al.* (55) and/or hydration. Both situations are possible, but we would favor the simplest one, namely that it catalyzes only the ring opening. As the subsequent reactions are too fast to be observed even without enzyme catalysis (8), it will be problematic to experimentally prove that the enzyme accelerates their rates. Since the spontaneous reaction is efficiently catalyzed by the hydroxide anion (Fig. 6), we propose that KdgF enzymes act *via* a similar base-catalyzed mechanism. Hence, a single catalytic residue acting as an acid-base could be sufficient. We propose Asp102 as this general acid-base, supported by structural evidence (Fig. 14, *A* and *B*), p*K*_a_ measurements by NMR, the pH activity profile of the enzyme and earlier mutational studies (55). Indeed, the homologous Asp100Ala mutant of *Ye*KdgF leads to a complete loss of activity (55). Moreover, the carboxylate group of Asp102 has a p*K*_a_ of 5.9 (Fig. 12), consistent with the optimum at about pH 7 of the activity of *Be*KdgF towards Δ (Fig. 9*A*). No other residue appears to have an ionizable group with a p*K*_a_ about 6, the closest being Asp110 and Glu66 having p*K*_a_ of 5.5 and 5.6, respectively (Table S4), which are both situated away from the active site. While the instability of Δ prevented co-crystallization, Δ was modeled and refined in the position of glycerol observed in the *Be*KdgF structure (Fig. 14*B*). The C1, O1, C2, and O2 of the modeled Δ superimposed well with the glycerol molecule in the crystal structure of *Be*KdgF (Fig. 14, *A* and *B*). The carboxylate of the Δ molecule also superimposed well with the best-defined carboxylate group of citrate in the active site of the *Ha*KdgF structure (<0.3 Å between the two carbons, PDB 5FQ0), facing potential charge stabilization by either Arg108 or an eventual metal cation. Notably, the modeled Δ molecule showed no steric clashes within the active site and appeared perfectly poised for a base-catalyzed reaction involving Asp102. Indeed, distances of 3.1 and 2.9 Å are observed between the anomeric O1 and the intracyclic O5 oxygen, respectively, and the Asp102 carboxylate for this modeled complex. Altogether, these mutational, structural, kinetic and NMR pH titration results make us assume that Asp102 acts as the catalytic base in *Be*KdgF. This leads to a proposal of a simple enzymatic mechanism for the ring opening catalyzed by *Be*KdgF leading to the linearized aldehyde (DEH) (Fig. 14*C*), which would leave the active site, becoming hydrated and cyclize into furanose form after keto-enol tautomerization (Fig. 7).

## Conclusion

*B. eggerthii* of the HGM requires cross-feeding to grow on alginate. Its three enzymes, the G-block specific *exo*-acting − potentially also *endo*-acting − *Be*PL6, the M-block specific *exo*-acting *Be*PL17, and the metalloprotein *Be*KdgF, catalyzing ring-opening of the 4,5-unsaturated monouronate product (Δ), were encoded by the same PUL and shown to cooperate in the utilization of alginate. A simple mechanism is proposed for *Be*KdgF catalyzed ring-opening of Δ, which was about 8-fold faster than the spontaneous conversion. Metal ions play a role in *Be*KdgF activity and thermostability. In light of the need for more insights into the deployment of natural resources in a sustainable way, the present findings on the 3 *B. eggerthii* enzymes can be important for future utilization of alginates and biomass.

## Experimental procedures

### Materials

*B. eggerthii* DSM 20697 (referred to as *B. eggerthii*) was purchased from Deutsche Samlung von Mikroorganismen und Zellkulturen. Brain Heart Infusion broth and LB broth were purchased from Sigma-Aldrich. Alginate ($\bar{M}$_n_ = 40 kDa, M/G ratio = 0.6) was a kind gift of Dr F. Madsen (DuPont, Nutrition and Health Denmark). PolyM ($\bar{M}$_w_ = 3 kDa, F_G_ = 0.0) was obtained from an epimerase-negative AlgG mutant of *Pseudomonas fluorescens* (65). PolyG ($\bar{M}$_w_ = 6‒8 kDa, F_G_ = 0.93) was prepared as previously described (89).

### Identification of alginate-degrading gut bacteria

The PULDB (38) was searched for *Bacteroides* strains from human fecal samples encoding at least two enzymes from CAZy PL families known for activity on alginates (39). *B. eggerthii* fulfilling these criteria was chosen for the present study.

### Growth of *B. eggerthii* on selective medium

*B. eggerthii* was grown in modified YCFA medium (see Supporting information) containing 0.5% (w/v) glucose overnight at 37 °C under anaerobic conditions (Whitley DG250 anaerobic workstation). Medium (8 ml) without carbon source (negative control), 0.5% (w/v) glucose (positive control), 0.5% alginate or 0.5% alginate with respective alginate lyases (100 nM A1-I, 100 nM *Be*PL6 or 100 nM *Be*PL17) was inoculated to a starting OD_600_ of 0.01 with *B. eggerthii* from an ON culture. The cultures were grown at 37 °C under anaerobic conditions (80% N_2_, 10% CO_2_ and 10% H_2_ from Air Liquide) and growth was monitored over 14 h by the absorbance at 600 nm.

### Isolation of genomic DNA

*B. eggerthii* was grown overnight at 37 °C under anaerobic conditions as above in 5 ml Brain Heart Infusion medium containing haemin (5 μg mL^‒1^). Cells from 1 ml culture were harvested by centrifugation (3200*g*, 4 °C, 10 min; Eppendorf 5810 R centrifuge), and genomic DNA was isolated using PureLink Microbiome DNA Purification Kit (Invitrogen) according to the manufacturer’s specifications. DNA concentration and quality were determined at 260 nm/280 nm (≥1.8) using NanoDrop (ThermoFisher).

### Amplification and cloning of genes

Genes encoding *Be*PL6, *Be*PL17 and *Be*KdgF were amplified from genomic DNA using either Phusion High-Fidelity DNA polymerase (*Be*PL6) or a modified Phusion High-Fidelity DNA polymerase (*Be*PL17 and *Be*KdgF). Primers were designed for restriction site cloning using NdeI and BamHI (*BePL6*) or USER cloning (*Be*PL17 and *Be*KdgF) (Table S1). Nucleotides encoding signal peptides in *Be*PL6 and *Be*PL17 predicted by SignalP (58) were omitted in the amplicons. *Be*PL6 was cloned into pET28a+ and *Be*PL17 and *Be*KdgF into the pET15b-USER vector. The *Be*PL6 construct was extended with an N-terminal His-tag and thrombin cleavage site (MGSSHHHHHHSSGLVPRGSH), while *Be*PL17 and *Be*KdgF constructs had an N-terminal His-tag (MGSSHHHHHHGS). The resulting plasmids were verified by sequencing (GATC Biotech). The gene encoding the alginate lyase A1-I from *Sphingomonas* sp. strain A1 was purchased codon optimized for *Escherichia coli* with an N-terminal His-tag from Genscript, cloned into pET-28a(+) by the restriction sites NheI and BamHI.

### Production and purification of recombinant enzymes

Plasmids encoding *Be*PL6, *Be*PL17, *Be*KdgF, and A1-I were heat shock transformed into *E. coli* BL21 DE3 then grown on agar plates containing kanamycin (*Be*PL6 and A1-I) or ampicillin (*Be*PL17 and *Be*KdgF). Single colonies were cultured in LB-KAN/AMP medium overnight, inoculated (10 ml) into 1 L LB medium containing ampicillin or kanamycin (final concentration 50 μg mL^−1^), and grown to OD_600_ 0.6‒0.8 (37 °C, 150 rpm). Expression was induced by isopropyl β-d-thiogalactopyranoside (IPTG) (0.5 mM) and followed by incubation (22 °C, 16 h). Cells were harvested by centrifugation (5000*g*, 4 °C, 20 min) and stored at −20 °C. Pellet corresponding to one-third L culture was resuspended in 20 ml 50 mM HEPES, 300 mM NaCl, pH 7.7, lysed (Pressure cell homogenizer, Stansted Fluid Power) and centrifuged (20,000*g*, 4 °C, 20 min). The supernatant was gently mixed (4 °C, 30 min) with 2 ml HisPur nickel-nitrilotriacetic acid resin (Thermo Fisher Scientific), pre-equilibrated in 20 ml 50 mM HEPES, 300 mM NaCl, pH 7.7. The resin was washed with 20 ml 50 mM HEPES, 300 mM NaCl, pH 7.7 and 20 ml 20 mM imidazole, 50 mM HEPES, 300 mM NaCl, pH 7.7, followed by elution by 10 ml 300 mM imidazole in the same buffer. The *Be*KdgF eluate was gel-filtered on Hi-load Superdex 75 26/60 and *Be*PL6, *Be*PL17, and A1-I eluates on Hi-load-Superdex 200 26/60 (GE Healthcare) in 50 mM HEPES, 150 mM NaCl, pH 7.7 at a flow rate of 2 ml min^‒1^. Protein purity was assessed by SDS-PAGE with 4 to 20% Mini-PROTEAN TGX Precast Protein Gels from Bio-rad stained with Coomassie and concentrations were determined spectrophotometrically at 280 nm using extinction coefficients predicted by ProtParam (62), ε_*Be*PL6_, ε_*Be*PL17_, ε_*Be*KdgF_ and ε_A1-I_ of 74,720, 106,580, 9970 and 108,095 M^‒1^ cm^‒1^. All purification steps were performed at 4 °C. From 800 ml LB media was obtained approximately 7.2 g, 9.1 g, 8.9 g, and 9.2 g BL21(DE3) cells containing *Be*PL6, *Be*PL17, and *Be*KdgF, respectively, yielding approximately 2.3 mg *Be*PL6, 1.7 mg *Be*PL17, 5.3 mg *Be*KdgF and 2.6 mg A1-I purified enzyme per gram of cells.

### Enzymatic characterization of *Be*PL6 and *Be*PL17

Optimal pH and salt concentration for *Be*PL6 and *Be*PL17 activity were determined using 20 mM UB4 buffer (90) pH 4‒9 and 0‒400 mM NaCl. The dependence was not analyzed at higher salt concentrations due to turbidity at >500 mM NaCl. Alginate was dissolved in the respective buffers to 2 mg mL^‒1^ and 200 nM *Be*PL6 and 100 nM *Be*PL17 were used in the assays. Substrate specificities of *Be*PL6 and *Be*PL17 were assessed using 0.38 mg mL^‒ 1^ alginate, polyG, and polyM as substrates and 100 nM enzyme in 50 mM HEPES, 150 nM NaCl, pH 7.7. Kinetics were determined using 0.19 to 5 mg mL^‒1^ substrate and 100 nM enzyme. The activity of *Be*PL6 was assayed in 50 mM HEPES, 0 M NaCl, pH 8.0, and of *Be*PL17 in 50 mM HEPES, 150 mM NaCl, pH 6.75 at 37 °C using 96-well UV-star chimney well plate (In Vitro, Australia). Enzyme and substrate were incubated separately (5 min, 37 °C), and mixed and the formation of 4,5-unsaturated uronate was monitored spectrophotometrically at 235 nm (56, 91) by measurements every 10 s using a plate reader (Bio-Tek Powerwave XS; Holm and Halby, Denmark). Initial velocities were obtained by linear regression analysis (GraphPad Prism) and product concentration was calculated using ε = 6150 M^‒1^ cm^‒1^ for the 4,5-unsaturated non-reducing end residues and monomeric Δ (92, 93, 94). The Michaelis-Menten model v_0_ = V_max_/(1+(*K*_m_/[S]_0_)) (95) was fitted to the initial velocities and substrate concentrations, and kinetic parameters were calculated using GraphPad Prism 9.3.1 (GraphPad Software). All experiments were done in triplicate and the standard deviation was plotted.

Cooperative degradation was determined on 0.5 mg mL^−1^ alginate in 50 mM HEPES, 150 mM NaCl, pH 7.7 at 37 °C using 100 nM for each of the four enzymes (*Be*PL6, *Be*PL17, *Be*KdgF, and A1-I) in the different combinations, monitored spectrophotometrically at 235 nm as above (56, 91) every 15 s during 4 h.

### Product analysis by LC-ESI-MS

Mixtures of 100 nM *Be*PL6 or *Be*PL17 and 5 mg mL^‒1^ alginate, polyM, or polyG were incubated (37 °C, 600 rpm mixing) in 50 mM HEPES, 150 mM NaCl, pH 7.7, and the reaction terminated at 0, 1, 2, 5, 10, 30 and 60 min by heat-inactivation for 10 min. Prior to analysis, samples were diluted 1:1 (v/v) in acetonitrile and centrifuged (10,000*g*, 4 °C, 10 min). Identification and relative quantification of alginate mono-, di- and oligosaccharides products was performed by liquid chromatography-electrospray ionization mass spectrometry (LC-ESI-MS) on an Amazon SL ion trap (Bruker Daltonics, Bremen Germany) coupled to an UltiMate 3000 UHPLC equipped with GlycanPac AXH-1 column, 150 × 2.1 mm (Thermo Fisher Scientific) as described previously (86). Relative quantitation of compound intensities was performed in TASQ 2.2 (Bruker Daltonics). DP1 products entail uronic acid monomers as well as DEH, DEH hydrates, and DHF epimers, which cannot be distinguished by LC-ESI-MS.

### SEC-MALS

Purified *Be*KdgF in 50 mM HEPES, 150 mM NaCl, pH 7.7 was concentrated to 1 mg mL^‒1^ using a spin column (3.5 kDa cut-off) and kept at 4 °C until analyzed. Sample (40 μl) was transferred to an HPLC autosampler vial and applied to the SEC-MALS instrument with a DAWN8+ detector (WYATT Technologies) connected to a Superdex 200 Increase 10/300 Gl column (GE Healthcare, USA) equilibrated with 50 mM HEPES, 150 mM NaCl, pH 7.7 eluted at a flow rate of 0.5 ml min^‒1^ using ice-cold buffer. Data were collected and analyzed using the built-in software (ASTRA, WYATT Technologies).

### *Be*KdgF mode of action and kinetics

Production of Δ for determination of linearization rate, pH optimum, and kinetics of *Be*KdgF was done just prior to use by mixing 400 nM *Be*PL17 and 4 mg mL^−1^ polyM in 50 mM HEPES, 150 mM NaCl, pH 7.0. At the time point showing ΔAbs_235_/dt = 0, pH was lowered to 3 by adding 6 M HCl, and *Be*PL17 was removed by filtration (Amicon Ultra Centrifugal filters, 10 kDa, Merck Millipore, Germany). The spontaneous linearization rate of Δ was determined by monitoring loss of absorbance at 235 nm (as above) in the range pH 1‒8 for 200 min followed by fitting a one-phase decay model to the data using GraphPad Prism 9.3.1 (GraphPad Software) and calculating the conversion rate constant and half-life. The one-phase decay model accounts for a combination of Δ undergoing ring opening and contraction according to first-order kinetics, and unsaturated AOS constituting an absorbance plateau over time.

Dose dependence of *Be*KdgF on activity was determined in a coupled assay, where Δ was produced by 100 nM *Be*PL17 acting on 0.5 mg mL^‒1^ polyM in 50 mM HEPES, 150 mM NaCl, pH 7.7 at 37 °C. At the time point where ΔAbs_235_/dt = 0, *Be*KdgF was added to final concentrations in the range of 0‒200 nM, and absorbance was monitored at 235 nm as above. Linearization rate constant and half-life were calculated as above.

The pH optimum of *Be*KdgF activity was determined spectrophotometrically at 235 nm in the pH range 5.5−8.0 (50 mM MES, 150 mM NaCl pH 5.5−7.0; 50 mM HEPES, 150 mM NaCl pH 6.5−8.0) for 0, 10 and 30 nM *Be*KdgF by measuring the rate of linearization of Δ produced from polyM by *Be*PL17 as above. The apparent rates were plotted against enzyme concentration to obtain the rate of enzyme catalysis, excluding the contribution from spontaneous linearization by subtracting the rate measured under identical conditions without *Be*KdgF added. Experiments were done in triplicate and all data points were plotted with standard deviation.

Δ produced as above was used for kinetics analysis in 50 mM HEPES, 150 mM NaCl, pH 7.0. Initial concentrations of Δ were determined from the absorbance at 235 nm using ε = 6150 M^‒1^ cm^‒1^ (91, 92, 96). The ring opening of Δ was measured without and with 20 nM *Be*KdgF and the contribution of the spontaneous linearization was subtracted. The Michaelis-Menten model was fitted to the data and a linear regression using GraphPad Prism8. Estimation of *k*_cat_/*K*_m_ was obtained by fitting the rate of reaction at low substrate concentrations to a linear correlation.

### *Be*KdgF metal ion dependence

For the following single-use utensils were used and or all glassware used was treated with 1 M HCl overnight, before thorough rinsing with ddH_2_O (97, 98, 99). *Be*KdgF was dialyzed against 50 mM HEPES, 50 mM EDTA, pH 7.0 (3 × 100-fold dilution, 4 °C, 3.5 kDa cut-off; SpectrumLabs, Greece) overnight to remove bound divalent cations, followed by dialysis against 50 mM HEPES, 150 mM NaCl, pH 7.0 (3 × 100-fold dilution) overnight. *Be*KdgF was diluted in 50 mM HEPES, 150 mM NaCl, pH 7.0, and divalent cations were added to appropriate samples to a final reaction concentration of 1 mM and incubated for 15 min at room temperature. EDTA was added to appropriate samples prior to assaying to final concentrations in the assay ranging from 0.1 to 1 mM. The metal dependence and effect of EDTA were assessed by monitoring the rate of conversion as described above. Δ was produced (approx. 450 μM) from 0.5 mg mL^‒1^ polyM using *Be*PL17 as described above.

### Thermal stability

The thermal stability of 2 μM *Be*PL6 and *Be*PL17 and 130 μM *Be*KdgF and *Be*KdgF in presence of 1 mM divalent metal ions as well as ZnCl_2_ in the range 0‒1 mM was analyzed in 50 mM HEPES, 150 mM NaCl, pH 7.0 using Prometheus Panta (Nanotemper) and Prometheus NT.48 capillaries. The samples were scanned from 15 to 90 °C at a rate of 1 °C min^–1^, intrinsic fluorescence being excited at 280 and emission monitored at 330 and 350 nm.

### Crystal structures of *Be*KdgF

*Be*KdgF (14.1 mg mL^‒1^ in 50 mM HEPES, 150 mM NaCl pH 7.7) was crystallized in 80 mM MES at pH 6.5 containing 14.4% (w/v) PEG8000, 20% v/v glycerol and 160 mM calcium acetate. In addition, *Be*KdgF in the same buffer was dialyzed at 4 °C against 50 mM EDTA using 100-fold excess of buffer changed twice with 3 h intervals, followed by overnight dialysis. After the metal depletion, the protein was dialyzed back into the original buffer in the same way (100-fold excess buffer, two buffer changes at 3 h intervals, followed by overnight dialysis at 4 °C). The dialyzed sample was concentrated to 14.1 mg mL^‒1^ and supplemented with 2.5 mM ZnCl_2_ and 2.5 mM guluronic acid immediately prior to setting up drops. Crystals formed in 200 mM potassium formate pH 7.3 containing 20% (w/v) PEG3500. Sitting drops containing equal volumes (150 nl) of protein and reservoir solutions were set up with a Gryphon liquid handling robot (Art Robbins Instruments) and equilibrated against 60 μl reservoir solution. Crystals with a dimension of approximately 100 μm formed within 1 day. MicroMount loops (MiTeGen) were used to harvest crystals which were flash frozen in liquid nitrogen, prior to which crystals from conditions supplemented with ZnCl_2_ and guluronic acid were cryo-protected by mixing 3 μl reservoir solution with 1 μl 87% glycerol and adding 1 μl of this solution to the 150 nl drop. For the *Be*KdgF sample without ZnCl_2_, a data set of 800 frames (0.2 degrees per frame) was collected at the BioMAX beamline (MaxIV, Lund, Sweden) with a detector distance of 254.15 mm and X-ray wavelength of 0.9677 Å. For the sample with ZnCl_2_, a data set of 2000 frames (0.1 degrees per frame) was collected at the P14 beamline (EMBL, Hamburg, Germany) with a detector distance of 305.2 mm and X-ray wavelength of 0.9763 Å. The data were processed using XDSAPP (100, 101, 102) and phased by molecular replacement using Phaser (103) in the Phenix software package (104), with the search model *Ye*KdgF (55) from *Y. enterocolitica* (PDB: 5FPX). An initial model was built with Phenix.autobuild (105) and completed with alternating manual rebuilding in Coot (106) and automatic refinement in Phenix.refine (105). The structure comparison extension of Phenix (104) was used to aid in structural comparison, and figures were generated using The PyMOL Molecular Graphics System, Version 2.5 Schrödinger, LLC. Statistics for the two models are reported in Table S2.

### NMR analysis of *Be*KdgF

All NMR spectra were recorded at 25 °C on a Bruker Avance III HD 800 MHz spectrometer using a 5 mm Z-gradient CP-TCI (H/C/N) cryogenic probe at the NV-NMR-Center/Norwegian NMR Platform (NNP) at the NTNU (Norwegian University of Science and Technology). The ^1^H signals were internally referenced to the water signal, and the ^13^C and ^15^N signals were indirectly referenced to the water signal based on absolute frequency ratios (107). All samples analyzed were ^13^C-^15^N-labelled *Be*KdgF (as described in (76)) in 90% H_2_O/10% D_2_O at enzyme concentrations in the range 0.26−1.04 mM (76). All NMR spectra were processed using TopSpin version 3.6.1.

Dynamic analysis was performed as described (77) using 150 μl *Be*KdgF (0.43 mM) in 25 mM Na_2_HPO_4_ (Merck) pH 7.2, 50 mM NaCl (VWR) in a LabScape Stream 3 mm NMR tube (Bruker). Nuclear spin relaxation times (T_1_, T_2_) and heteronuclear {^1^H}-^15^N NOE measurements of amide ^15^N for *Be*KdgF. T_1_ and T_2_ spectra were recorded as pseudo-3D spectra where two frequency dimensions correspond to the amide ^1^H and ^15^N chemical shifts, respectively, and the third dimension is made up of variable relaxation time delays. For T_1_, the time points were 0.1, 0.2, 0.5, 1, 1.5, 2, 2.5, 3, 3.5, 4 and 4.5 s. For T_2_, the time points were 17, 34, 68, 136, 170, 204, 238 and 272 ms. The ^1^H-^15^N NOE spectra were composed of two 2D planes recorded with and without pre-saturation, respectively. Overall rotational correlation times (τ_c_) were determined from the ratio between T_1_ and T_2_ (108). The obtained data were analyzed using the software Dynamics Center version 2.7.4.

A pH titration was performed of *Be*KdgF (0.26 mM, 500 μl) in 20 mM HEPES (ITW Reagents) pH 7.2, 25 mM NaCl (VWR) in a thin-walled 5 mm Ø SP Wilmad-LabGlass NMR tube (VWR). The pH values were adjusted by transferring the sample from the NMR tube to an Eppendorf tube, adding HCl (1 and 0.1 M, Merck) and/or NaOH (1 and 0.1 M, VWR) in small steps of ≈0.1 μl until the desired pH value was reached, and then transferring the sample back into the same NMR tube. At each pH value point, an ^15^N HSQC spectrum was recorded. The peak movements were measured using the CARA software version 1.8.4.2 (109). The *p*K_a_ values of the residues were calculated using the Henderson-Hasselbalch equation (79).

## Data availability

Structures presented in this work have been deposited to the Protein Data Bank (PDB) with the following PDB codes: 7ZYB and 7ZYC.

## Supporting information

This article contains supporting information (62).

## Conflict of interest

The authors declare that they have no conflicts of interest with the contents of this article.
