# Supplementary material for: Action and cooperation in alginate degradation by three enzymes from the human gut bacterium Bacteroides eggerthii DSM 20697
Source: J Biol Chem. 2024 Jul 19;300(9):107596. doi: 10.1016/j.jbc.2024.107596 (PMC11381880; doi:10.1016/j.jbc.2024.107596)
Supplement: Supporting information [file mmc1.pdf]

# Supporting information:

## Action and cooperation in alginate degradation by three enzymes from the human gut bacterium *Bacteroides eggerthii* DSM 20697

**Mette E. Rønne[1,3,5], Christian Dybdahl Andersen[1,5], David Teze[1,2], Agnes Beenfeldt Petersen[3], Folmer Fredslund[2], Emil G. P. Stender[1], Evan Kirk Chaberski[2], Jesper Holck[4], Finn L. Aachmann[3], Ditte Hededam Welner[2] and Birte Svensson[1*]**

*From [1]Enzyme and Protein Chemistry, Department of Biotechnology and Biomedicine, Technical University of Denmark, DK-2800, Kgs. Lyngby, Denmark; [2]Enzyme Engineering and Structural Biology, Novo Nordisk Foundation Center for Biosustainability, Technical University of Denmark, DK-2800 Kgs. Lyngby, Denmark; [3]Norwegian Biopolymer Laboratory (NOBIPOL), Department of Biotechnology and Food Science, NTNU Norwegian University of Science and Technology, N-7491 Trondheim, Norway; [4]Enzyme Technology, Department of Biotechnology and Biomedicine, Technical University of Denmark, DK-2800, Kgs. Lyngby, Denmark*

*[5]Equal contribution*

Note: Current addresses for CDA: Arla Foods Ingredients, Arinco, Mælkevejen 2-4, DK-6920 Videbæk, Danmark; DT: Department of Chemistry, University of Copenhagen, Universitetsparken 5, DK-2100 Copenhagen Ø, Denmark; EGPS: Fida Biosystems Aps, Generatorvej 6, DK-2860 Søborg, Denmark; EKC: River Stone Biotech ApS, Fruebjergvej 3, DK-2100 Copenhagen Ø, Denmark

Supporting information: Experimental procedure, Tables S1–S4 and Figures S1–S9.

## Experimental procedures

Modified YCFA medium: 2 g casitone, 0.8 g $NaHCO_3$, 90 mg $K_2HPO_4$, 90 mg $KH_2PO_4$, 180 mg NaCl were mixed with 140 mL MQ water. Stock solutions of vitamins were added to the medium, corresponding to 0.2 mg Resazurin, 2 µg biotin, 2 µg cobalamin, 6 µg *p*-aminobenzoic acid, 10 µg folic acid and 30 µg pyridoxamine. A solution of haemin (2 mg) was added, and the media is boiled for 5 min until the color changed to red. The medium is autoclaved for 15 min at 121°C along with separate bottles containing 2% w/v solutions of appropriate carbon sources (glucose (positive control), water (negative control) and alginate). The medium was cooled to room temperature and solutions of cysteine (0.2 g), $CaCl_2$ (18 mg), $MgSO_4{\cdot}7H_2O$ (18 mg), thiamin (10 µg) and riboflavin (10 µg) were added using sterile filtrations to a final volume of 150 mL. Appropriate carbon source was added to a final concentration of 0.5% (w/v) under sterile conditions and pH was adjusted to pH 7.3.

# Tables

**Table S1**

**Primers for amplification of selected genes from *Bacteroides eggerthii* DSM 20697.** All primers were designed to have a nonsense overhang (six bp) in the 5’ end and the restrictions sites Ndel (Forward primer) and BamHI (Reverse primer) over the nonsense overhang

| Protein encoded by gene | Primers | Annealing temperature [°C] | Extension time [s] |
|---|---|---|---|
| *Be*PL6 | Fwd: 5’ GAT GCT CAT ATG GCA TGT TCG CAA GGA AAT TG 3’<br>Rev: 5’ GAT GCT GGA TCC TTA TGT GAT TCC TAA ATC TTT CTT GTC 3’ | 61 | 60 |
| *Be*PL17 | Fwd: 5’ GAT GCT CAT ATG AAG TTA CCA GAG CAT CCC 3’<br>Rev: 5’ GAT GCT GGA TCC TTA TTT TAT TTC GCA GAC TCC TT 3’ | 59 | 60 |
| *Be*KdgF | Fwd: 5’ GAT GCT CAT ATG AAA ACA TGT AGT AAA GTT TTT C 3’<br>Rev: 5’ GAT GCT GGA TCC TTA TTC GTT TAT AAA ATC TTC ACG 3’ | 57 | 20 |

**Table S2**
**Data collection and refinement statistics of *Be*KdgF-Ca and *Be*KdgF-Zn.** Statistics for the highest-resolution shell are shown in parentheses

| Protein | *Be*KdgF-Ca | *Be*KdgF-Zn |
|---|---|---|
| PDB code | 7ZYB | 7ZYC |
| **Data collection** | | |
| Beamline | P-14 EMBL | MaxIV-Biomax |
| Wavelength (Å) | 0.9677 | 0.9763 |
| Resolution range (Å) | 41.0–1.5 (1.55–1.5) | 40.4–2.0 (2.07–2.0) |
| Space group | $P6_222$ | $P6_222$ |
| Unit cell a=b, c (Å) | 47.37, 175.86 | 46.66, 176.96 |
| Total No. of reflections | 261211 (8914) | 166949 (12546) |
| No. of unique reflections | 19628 (1845) | 8420 (799) |
| $R_{merge}$ | 0.052 (2.167) | 0.083 (1.549) |
| $R_{meas}$ | 0.055 (2.431) | 0.085 (1.6) |
| $R_{pim}$ | 0.014 (1.069) | 0.019 (0.395) |
| $CC_{1/2}$ | 0.999 (0.484) | 1 (0.921) |
| $CC^*$ | 1 (0.808) | 1 (0.979) |
| $I/\sigma(I)$ | 20.91 (0.62) | 23.56 (1.80) |
| Completeness (%) | 98.85 (95.32) | 98.85 (97.90) |
| Multiplicity | 13.3 (4.8) | 19.8 (15.7) |
| **Refinement** | | |
| Reflections used in refinement | 19546 (1833) | 8339 (791) |
| Reflections used for R-free | 944 (93) | 837 (79) |
| $R_{work}$ | 0.174 (0.352) | 0.228 (0.464) |
| $R_{free}$ | 0.201 (0.367) | 0.260 (0.544) |
| *CC* (work) | 0.973 (0.756) | 0.952 (0.854) |
| *CC* (free) | 0.977 (0.763) | 0.926 (0.371) |
| Number of non-hydrogen atoms | 1001 | 888 |
| Macromolecules | 931 | 872 |
| Ligands | 16 | 8 |
| Solvent | 62 | 8 |
| Average *B*-factor | 40.88 | 69.96 |
| Macromolecules | 40.61 | 70.04 |
| Ligands | 51.44 | 74.44 |
| Solvent | 43.52 | 57.04 |
| Wilson B-factor | 25.42 | 42.45 |
| Protein residues | 112 | 111 |
| r.m.s.d | | |
| Bonds (Å) | 0.022 | 0.009 |
| Angles (˚) | 1.79 | 0.87 |
| Ramachandran plot | | |
| Favored (%) | 97.27 | 99.08 |
| Allowed (%) | 2.73 | 0.92 |
| Outliers (%) | 0.00 | 0.00 |
| Rotamer outliers (%) | 3.92 | 2.11 |
| Clashscore | 4.83 | 2.88 |
| Number of TLS groups | 9 | 3 |

**Table S3**
**B-factor comparison of conserved metal-coordinating residues relative to global B-factor in *Be*KdgF and the two homologs**

| **Residue B-factor relative to global B-factor** | | | | | | | |
|---|---|---|---|---|---|---|---|
| *Be*KdgF | | | | Homologs | | | |
| Residue | *Be*KdgF-Ca | *Be*KdgF-Zn | | Residue | *Ye*KdgF (chain A) | Residue | *Ha*KdgF (chain A) |
| His50 | 1.18 | 1.25 | | His46 | 0.84 | His48 | 0.86 |
| His52 | 0.87 | 0.95 | | His48 | 0.89 | His50 | 0.77 |
| Gln56 | 0.70 | 0.87 | | Gln53 | 0.76 | Gln55 | 0.74 |
| His90 | 0.89 | 0.99 | | His87 | 0.81 | His89 | 0.80 |

**Table S4**
**p$K_a$ values of amino acid residues in *Be*KdgF titrating in the pH range 4.15-8.02**

| | **Glu13 ($^{15}$N)** | **Glu17 ($^{15}$N)** | **Asp21 ($^{15}$N)** | **Glu66 ($^{15}$N)** | **Glu66 ($^{1}$H)** |
|---|---|---|---|---|---|
| **p$K_a$** | 5.0 ± 0.2 | 4.9±0.1 | 5.0±0.2 | 5.6±0.1 | 5.1±0.2 |
| **δAH [ppm]** | 118.3±0.02 | 124.7±0.04 | 123.7±0.02 | 122.5±0.01 | 9.1±0.002 |
| **δA [ppm]** | 118.7±0.1 | 126.5±0.1 | 124.2±0.1 | 122.0±0.04 | 9.0±0.01 |

| | **Asp102 ($^{15}$N)** | **Asp110 ($^{1}$H)** | **Glu114 ($^{15}$N)** | **Glu114 ($^{1}$H)** |
|---|---|---|---|---|
| **p$K_a$** | 5.9±0.1 | 5.5±0.2 | 5.1±0.1 | 5.2±0.1 |
| **δAH [ppm]** | 128.1±0.02 | 9.1±0.002 | 127.0±0.04 | 7.9±0.002 |
| **δA [ppm]** | 128.6±0.04 | 9.2±0.01 | 128.4±0.01 | 8.0±0.004 |

# Figures

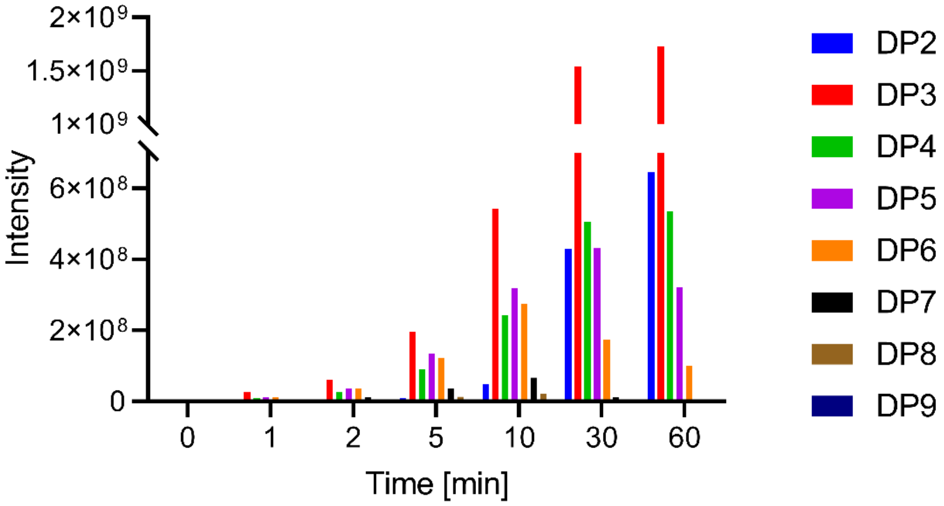


**Figure S1. Product formation by A1-I from *Sphingomonas* sp. A1 from alginate determined by LC-ESI-MS.** A) Product formation by 150 nM A1-I from 2.5 mg $mL^{-1}$ alginate in 50 mM HEPES, 150 mM NaCl, pH 7.7.

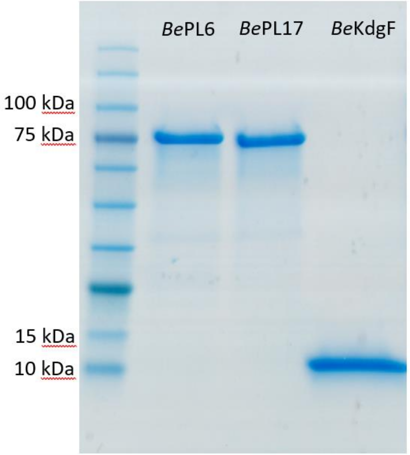


**Figure S2. SDS-PAGE of recombinant proteins**. *Be*PL6, *Be*PL17 and *Be*KdgF from *Bacteroides eggerthii* DSM 20697 were produced in *E. coli* and purified in two steps yielding 2.3, 1.7 and 5.3 mg per gram of BL21 DE3 cells, respectively. The three proteins migrated as single bands corresponding to the expected sizes predicted by ProtParam (1) to 84150.59 Da, 82093.94 Da and 14137.04 Da, respectively.

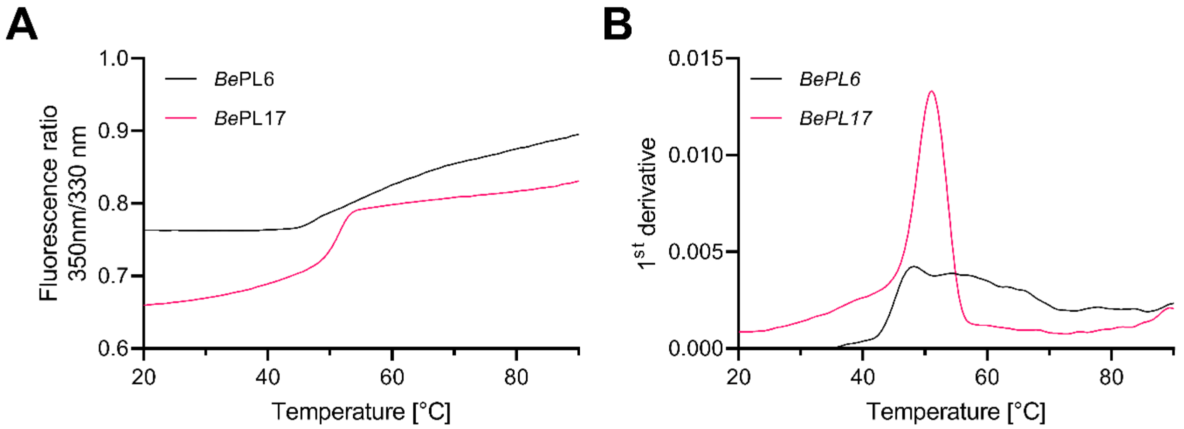


**Figure S3. Thermal denaturation curves.** A) Fluorescence ratio 350 nm/330 nm of *Be*PL6 and *Be*PL17. B) First derivative of thermal unfolding of *Be*PL6 and *Be*PL17. Thermal stability of 2 µM *Be*PL6 and *Be*PL17 in 50 mM HEPES, 150 mM NaCl pH 7.0 in Prometheus NT.48 capillaries and analyzed by Prometheus Panta (Nanotemper, Munich. Germany).

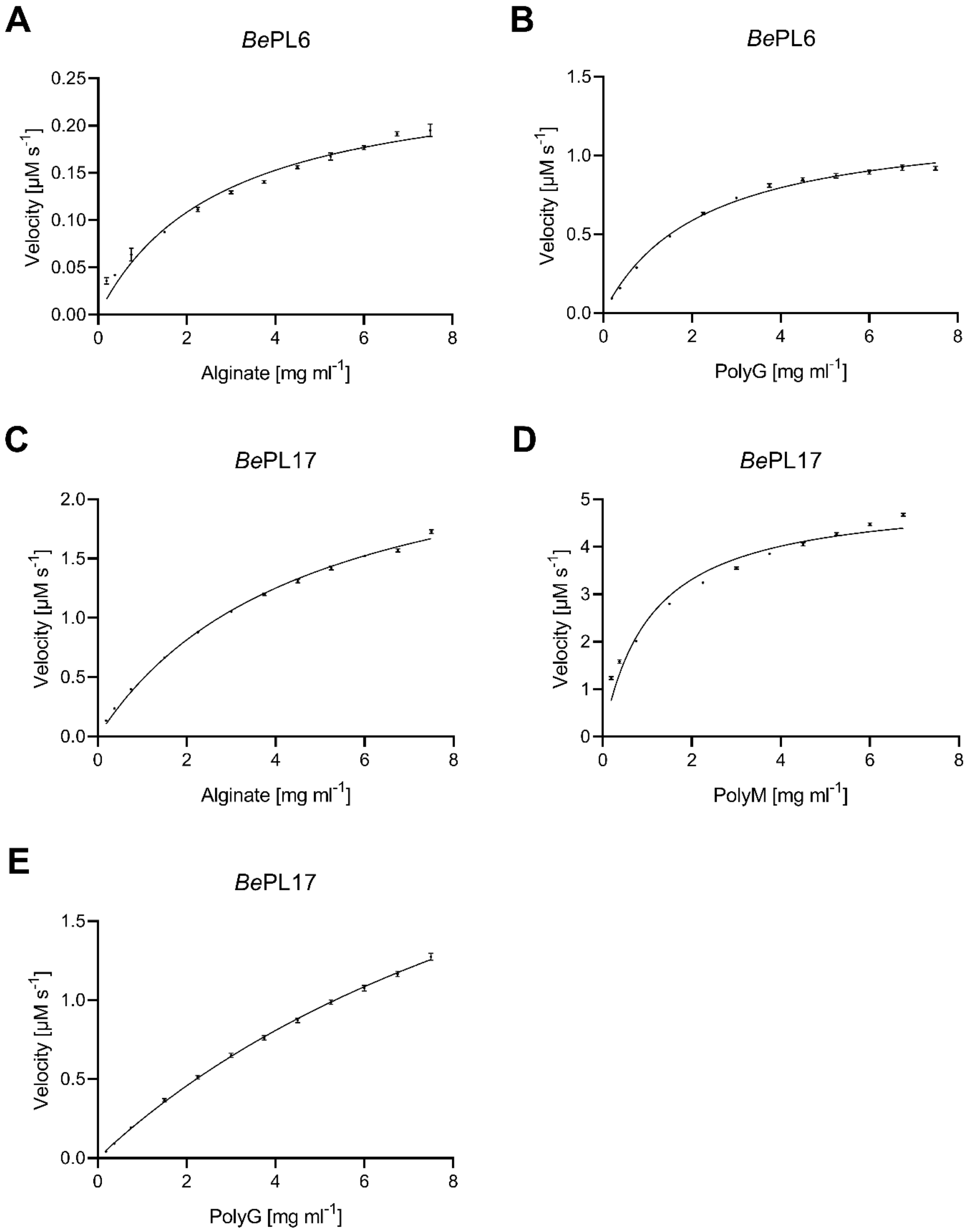


**Figure S4. Michaelis-Menten plots of *Be*PL6 and *Be*PL17.** The Michaelis-Menten model was fitted to Initial velocities vs. substrate concentrations using GraphPad Prism 9.3.1. A) 100 nM *Be*PL6 on alginate. B) 100 nM *Be*PL6 on polyG. C) 100 nM *Be*PL17 on alginate. D) 100 nM *Be*PL17 on polyM. E) 100 nM *Be*PL17 on polyG. Activity of *Be*PL6 was assayed in 50 mM HEPES, 0 M NaCl, pH 8.0 and of *Be*PL17 in 50 mM HEPES, 150 mM NaCl, pH 6.75. Samples are run in triplicate and presented as the mean ± SD.

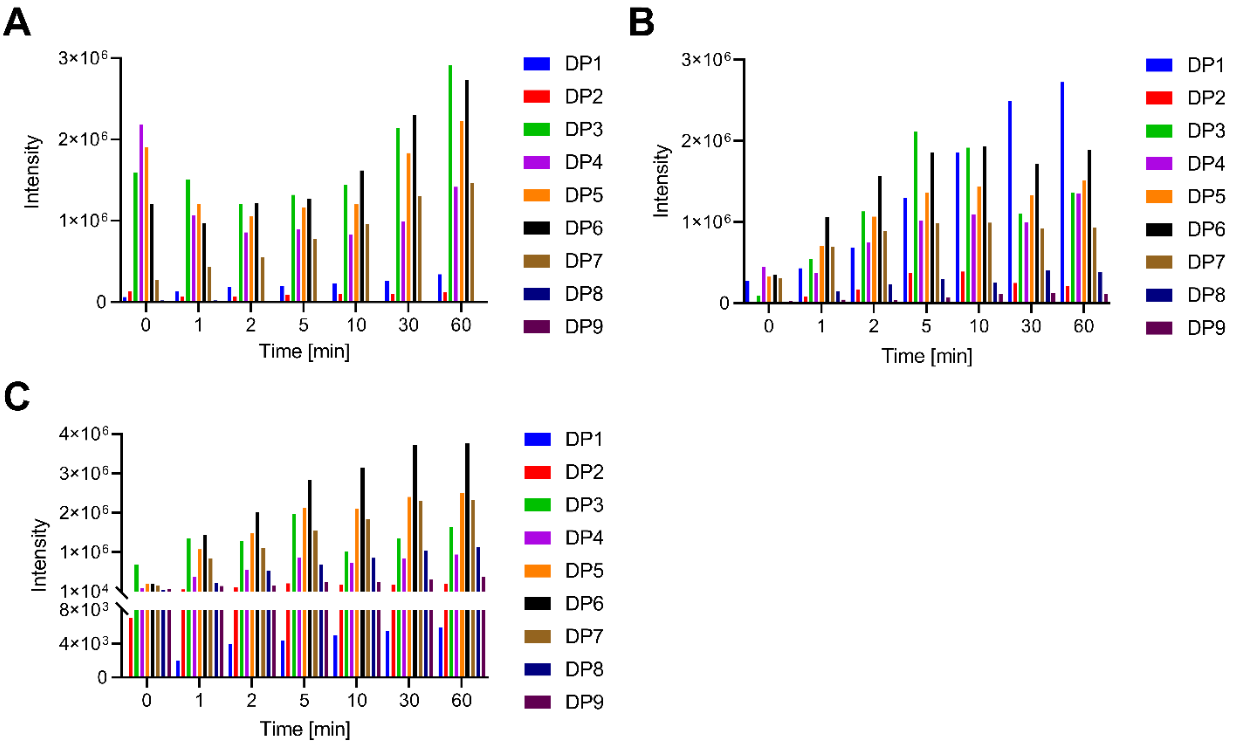


**Figure S5. Product analysis by LC-ESI-MS of *Be*PL6 and *Be*PL17**. A) Product formation by *Be*PL6 from alginate. B) Product formation by *Be*PL17 from alginate. C) Product formation by *Be*PL17 from polyG. Reaction performed in 50 mM HEPES, 150 mM NaCl, pH 7.7.

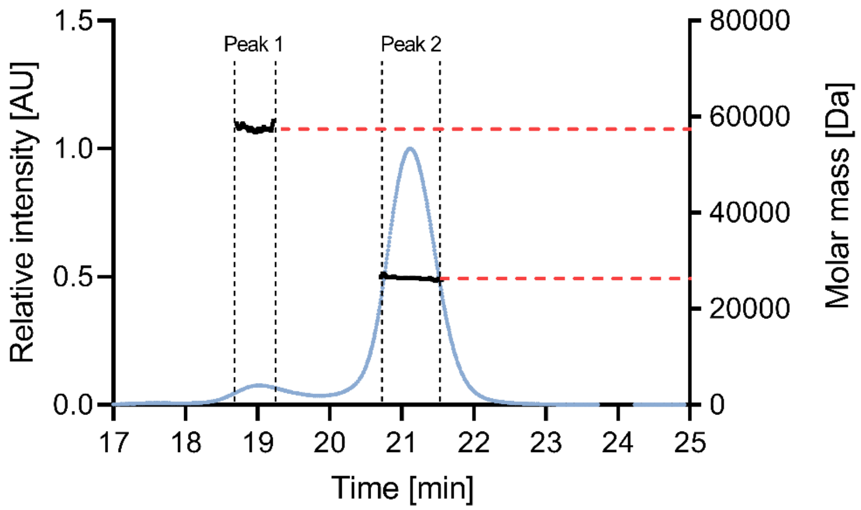


**Figure S6. SEC-MALS analysis of *Be*KdgF**. *Be*KdgF (40 µL, 1 mg mL$^{-1}$) in 50 mM HEPES, 150 mM NaCl, pH 7.7 was separated at 4°C at a flow rate of 0.5 mL min$^{-1}$ on a Superdex 200 Increase 10/300 GL column connected to a SEC-MALS instrument with a DAWN8+ detector.

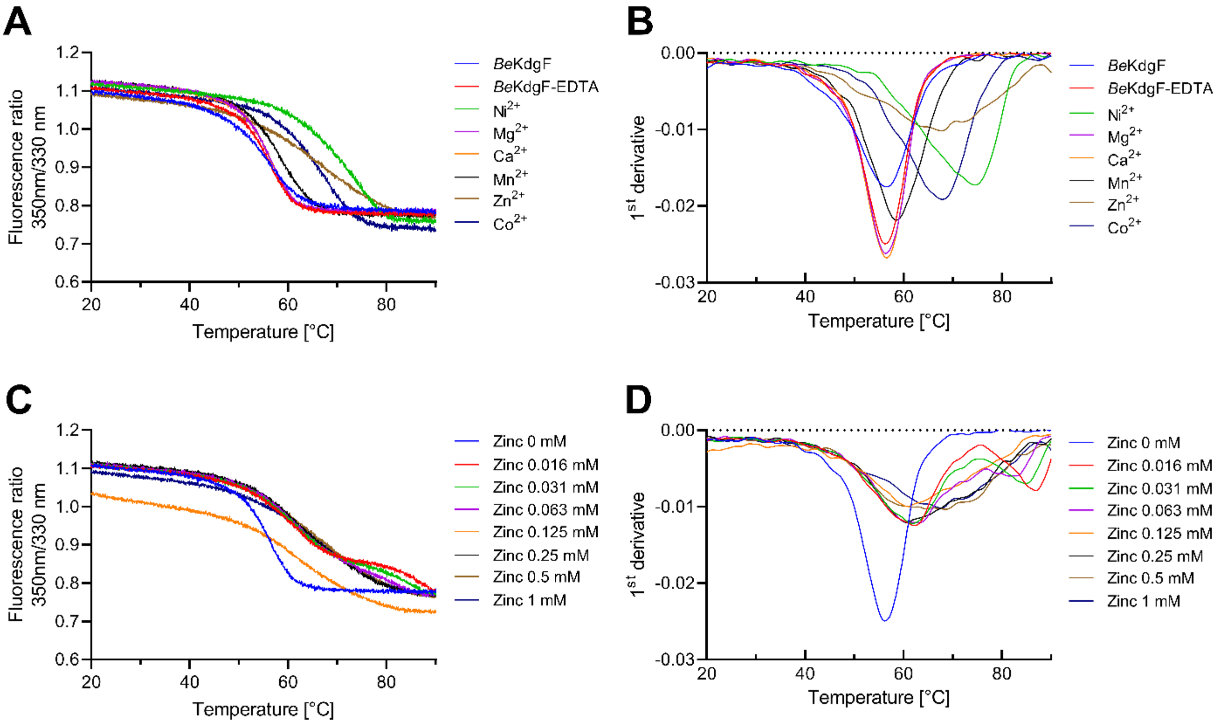


**Figure S7. Thermal denaturation curves.** A) Fluorescence ratio 350 nm/330 nm of *Be*KdgF with and without addition of divalent cations. B) First derivative obtained from data in A of thermal unfolding. C) Fluorescence ratio 350 nm/330 nm of *Be*KdgF with addition of increasing amounts of $Zn^{2+}$. D) First derivative obtained from data in C of thermal unfolding. Thermal stability of 130 µM *Be*KdgF in 50 mM HEPES, 150 mM NaCl, pH 7.0 in Prometheus NT.48 capillaries and analyzed by Prometheus Panta (Nanotemper, Munich, Germany).

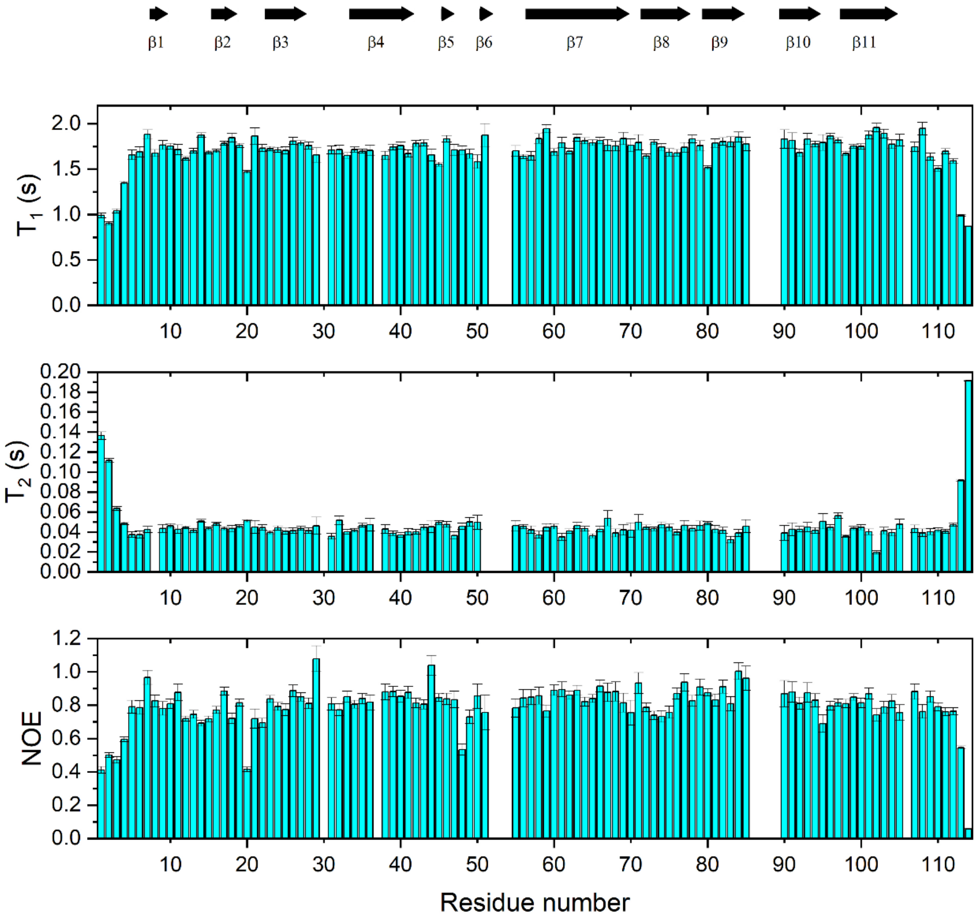


**Figure S8. Observed values of $^{15}$N $T_1$, $^{15}$N $T_2$, and {$^{1}$H}-$^{15}$N NOE in *Be*KdgF.** Measurements were performed at 800 MHz and 25 °C. The secondary structure of the protein is indicated above.

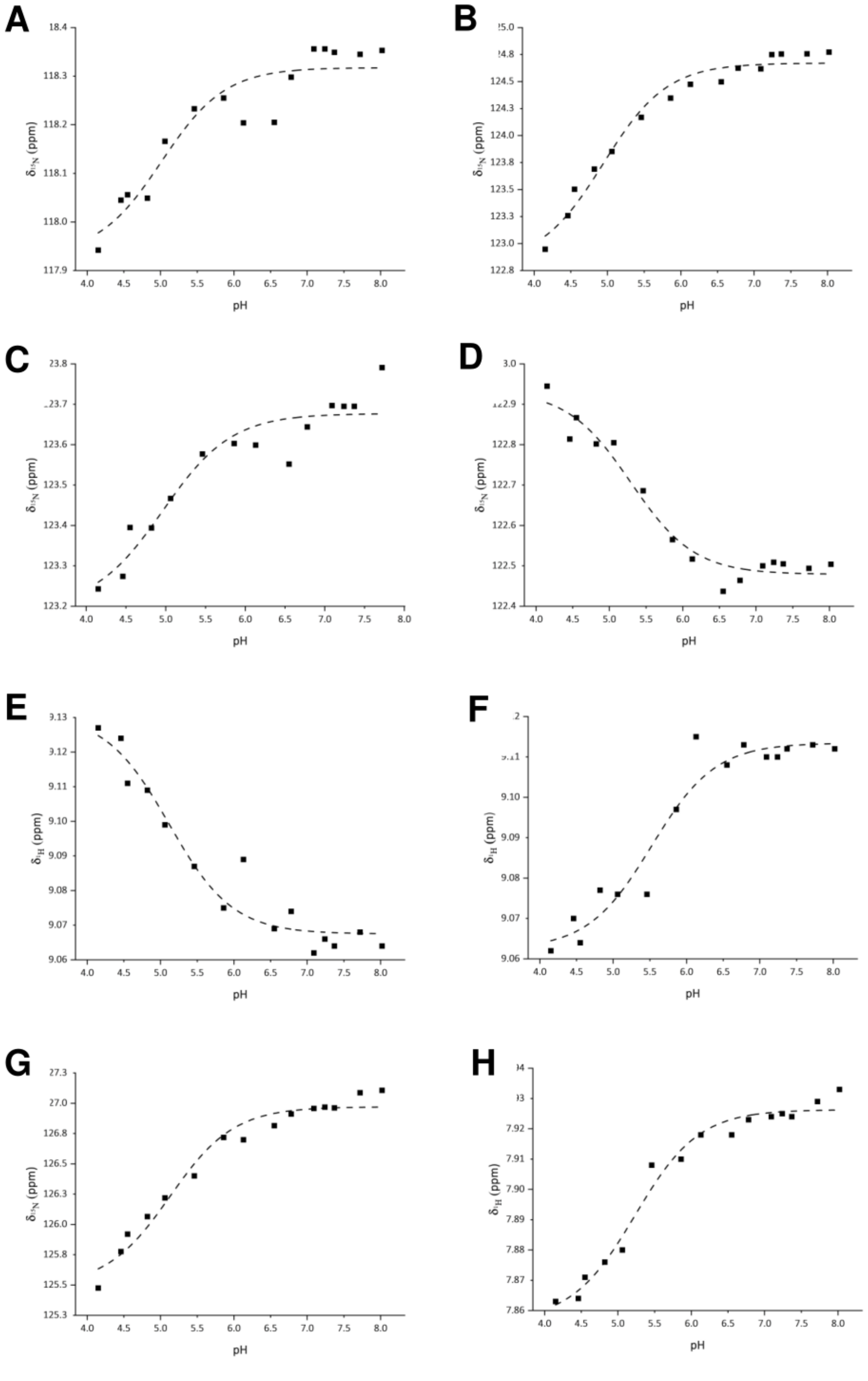


**Figure S9. Chemical shifts of residues in *Be*KdgF by pH titration from 4.15 to 8.02 observed by NMR**. A) Glu13 ($^{15}N$). B) Glu17 ($^{15}N$). C) Glu21 ($^{15}N$). D) Glu66 ($^{15}N$). E) Glu66 ($^{1}H$). F) Asp110 ($^{1}H$). G) Glu114 ($^{15}N$) and H) Glu114 ($^{1}H$).

# Reference

1. Gasteiger, E., Hoogland, C., Gattiker, A., Duvaud, S., Wilkins, M. R., Appel, R. D., and Bairoch, A. (2005) *Protein Identification and Analysis Tools on the ExPASy Server.*, Humana Press, Totowa, NJ
